# Risk factors for tibial infections following osteosynthesis – a systematic review and meta-analysis

**DOI:** 10.1016/j.jcot.2024.102376

**Published:** 2024-02-23

**Authors:** Diana Niebuhr, Thomas Mattson, Niels Martin Jensen, Bjarke Viberg, Signe Steenstrup Jensen

**Affiliations:** a Department of Orthopedic Surgery and Traumatology, Lillebaelt Hospital, Kolding, Denmark; b Emergency Department, Aarhus University Hospital, Aarhus, Denmark; c Department of Orthopedic Surgery and Traumatology, Lillebaelt Hospital, Vejle, Denmark; d Department of Orthopedic Surgery and Traumatology, Odense University Hospital, Odense, Denmark; e Institute of Regional Health Research, University of Southern Denmark, Odense, Denmark; f Department of Clinical Research, University of Southern Denmark, Odense, Denmark

**Keywords:** Surgical site infection, Fractures, Meta-analysis, Osteosynthesis, Risk factors

## Abstract

**Aim:**

This study aimed to quantitatively summarise risk factors associated with surgical site infection (SSI) following surgically managed tibial fractures.

**Method:**

We searched the Embase/Medline, Cochrane Library, and Scopus databases for relevant studies in October 2023. We included original studies investigating risk factors for SSI following surgically managed traumatic tibial fractures that included ≥10 adult patients with SSIs. Meta-analysis was performed when >5 studies investigated the same risk factor. The risk of bias was assessed using the critical appraisal checklist from Joanna Briggs Institute for cohort studies.

**Results:**

This study included 42 studies comprising 24,610 patients with surgically managed tibial fractures and 2,418 SSI cases. The following were identified as significant risk factors for SSI (p < 0.05): compartment syndrome (odds ratio [OR] = 3.8, 95% confidence interval [CI]: 2.4–6.0), blood transfusion (OR = 3.8, 95% CI: 2.1–6.6), open fracture (OR = 3.6, 95% CI: 2.5–5.1), Gustilo–Anderson classification >2 (OR = 3.1, 95% CI: 2.1–4.6), external fixation (OR = 2.9, 95% CI: 2.3–3.8), American Society of Anesthesiologists classification >2 (OR = 2.5, 95% CI: 1.5–4.1), polytrauma (OR = 2.4, 95% CI: 1.5–4.0), dual incision approach (OR = 2.1, 95% CI: 1.5–3.0), smoking (OR = 1.8, 95% CI: 1.5–2.1), male sex (OR = 1.6, 95% CI: 1.3–1.8), high energy trauma (OR = 1.5, 95% CI: 1.1–2.1), and prolonged surgery time (OR = 0.62, 0.43–0.82). Other factors, including diabetes, hypertension, and time to surgery, were not identified as risk factors for SSI. However, the included studies were generally of poor quality and at risk of bias.

**Conclusions:**

The review provides a basis for preoperatively assessing a patient's risk of developing an SSI, which could be used to initiate adjusted antibiotic regimes and more frequent postoperative controls. Furthermore, it indicates the risk factors future research should include when adjusting for confounding factors.

## Highlights

• Patient related risk factors for infection are male sex, ASA classification>2 and smoking.
• Fracture related risk factors are open fracture, Gustilo Anderson type, compartment syndrome, high energy- and polytrauma.
• Surgery related risk factors are external fixation, blood transfusions, prolonged operative time, and dual incision.
• This review provides basis for preoperative assessment of a patient’s risk for developing SSI.
• This review indicates which risk factors future research should include when assessing for confounding factors.

## 1 Introduction

Tibial fractures are adults’ most common long bone fractures, often requiring surgery to stabilize the bone.^1^ One of the most challenging and devastating complications after surgery for tibial fractures is surgical site infection (SSI).^2^ SSI management often involves repeated operations, prolonged hospital stays, and rehabilitation. It comes with significant expenses for the patient, whose quality of life and ability to work can be significantly affected, and it is very costly for the healthcare system.^3^ Therefore, preventing SSIs is essential.

The risk factors for SSI in tibial fractures have been investigated in numerous studies with divergent results.^4^, ^5^, ^6^, ^7^ Numerous studies have confirmed compartment syndrome, open fractures, external fixation, and tobacco use as risk factors for SSI. However, male sex, operative time, and alcohol use have shown mixed results.^4^^,^^5^^,^^8^^,^^9^ Two systematic reviews have investigated risk factors for SSI after open reduction internal fixation (ORIF) of tibial plateau^5^ and ankle^4^ fractures comprising 2,214 and 8,103 patients, respectively. They both showed that open fracture was a risk factor for SSI but had opposing results for external fixation, operative time, and smoking. No published study has compiled current knowledge which would help assess patient susceptibility to SSI and initiate preventive measures when required.

Therefore, this study aimed to quantitatively estimate risk factors associated with SSI following surgically managed tibial fractures.

## 2 Methods

### 2.1 Protocol and registration

This systematic review and meta-analyses are reported to Systematic Reviews and Meta-Analyses (PRISMA) statement according to the Preferred Reporting Items.^10^ The study protocol was registered in the International Prospective Register of Systematic Reviews (PROSPERO) database before data extraction (registration number: CRD42022324456).^11^

### 2.2 Eligibility criteria

The PECO model was used to create the research string:

**P**opulation: Patients with SSIs after osteosynthesis for traumatic tibia fractures.

**E**xposure: Risk factors associated with the development of SSIs.

**C**omparator: Patients who did not suffer from SSIs after osteosynthesis for traumatic tibia fractures.

**O**utcome: Patients with SSIs.

The inclusion criteria were peer-reviewed studies with patients aged >15 suffering from an open or closed traumatic tibial fracture treated with osteosynthesis.

The exclusion criteria were animal and cadaveric studies; studies with <10 patients developing an SSI after surgery^12^; studies where fractures were treated with prostheses or conservatively; studies on face, head, neck, thoracic, or spine fractures; studies involving cancer or tumor surgery; studies on periprosthetic fractures; studies on gunshot or explosive fractures; studies on arthrodesis; and studies published in languages other than English, Swedish, German, Norwegian, or Danish.

### 2.3 Definition of risk factors and infection

Infection was not defined before the screening process. All studies describing SSIs and investigating risk factors potentially affecting postoperative SSI risk for tibial fractures were included.

### 2.4 Information sources

We conducted a systematic literature search on 30 October 2023 in the Cochrane, Embase/Medline, Library, and Scopus electronic databases. Grey literature was sought on The European Bone and Joint Infection Society (https://ebjis.org; date: 30/10/2023) and European Wound Management Association (https://ewma.org; date: 30/10/2023) websites; no further studies were identified.

### 2.5 Search strategy

The search string comprised MeSH terms and free-text words in four blocks with the following synonyms; tibia, infection, fracture, and osteosynthesis. The Boolean operator “AND” combined the four blocks: “tibia AND infection AND fracture AND osteosynthesis”. In each block, the Boolean operator “OR” was used between synonyms. See Appendix A for the complete search string. No search limitations were included.

### 2.6 Study selection

All studies were imported into EndNote and searched for duplicates. Then, studies were imported into Covidence (Veritas Health Innovation, Australia; www.covidence.org) for screening. The included studies were screened against the inclusion and exclusion criteria independently and blindly by the two primary authors (DN and TM). The included studies were then full-text screened separately by the same two authors. Consultation with senior authors resolved any disagreements.

### 2.7 Data collection process

Relevant data were extracted independently by the two primary authors into a predesigned Excel sheet (Microsoft Excel for Mac, Office 365 version 16.44). We contacted 18 authors by email due to missing data (i.e., the number of patients in each exposure group). Five replied with a datasheet,^6^^,^^13^, ^14^, ^15^, ^16^ ten were excluded due to missing data, and three were included in the systematic review but excluded from meta-analysis due to missing raw data.^7^^,^^8^^,^^17^ Finally, the two primary authors double-checked all extracted data.

### 2.8 Data items

For each study following variables were registered: author name, publication year, study design, country, number of participants, number of infected patients, minimum follow-up, patient mean age, open/closed fractures, and number of patient-related risk factors.

### 2.9 Risk of bias in individual studies

Studies included in the meta-analysis were assessed for risk of bias using the Joanna Briggs Institute critical appraisal checklist for cohort studies.^18^ The first two studies were evaluated as a pilot and then blindly assessed by a senior author (BV) to ensure agreement. Then, the remaining studies were all assessed by one author and subsequently discussed with another author (DN + TM and SS + NJ, respectively).

Three senior authors selected four critical confounding factors known to affect the risk of SSI: age, diabetes, tobacco use, and open fracture. The outcome was always SSI and was considered reliable in cases with purulent discharge, fistula or wound breakdown, or local and systemic infection symptoms with an affected blood test. The outcome was assessed as ‘unclear’ if a surgeon noted infection. A minimum of 6 months of follow-up was defined as sufficient for an outcome to have occurred. ‘Not applicable’ was used in question 10 when a follow-up was deemed ‘unclear’ in question 9.

### 2.10 Statistics and synthesis of results

SSI and risk factors were assessed as binary outcomes, and a 2 × 2 contingency table was created for each risk factor. The Stata 16 (version 2019; StataCorp LLC, College Station, USA) software was used for all statistical analyses. We assume that fracture localization does not influence the risk of infection and therefore compile data from the entire tibia for meta-analysis. Meta-analyses were performed when a potential risk factor was investigated in ≥5 studies. The evidence synthesis was performed using an odds ratio (OR) as the effect measure, with a *p* < 0.05 considered statistically significant. The meta-analyses were conducted using the built-in Meta function in Stata 16 and reported as forest and funnel plots. Data were assessed using a random-effects model and restricted maximum likelihood (REML) due to the heterogenicity in data.^19^

## 3 Results

### 3.1 Study selection

**Fig. 1:**
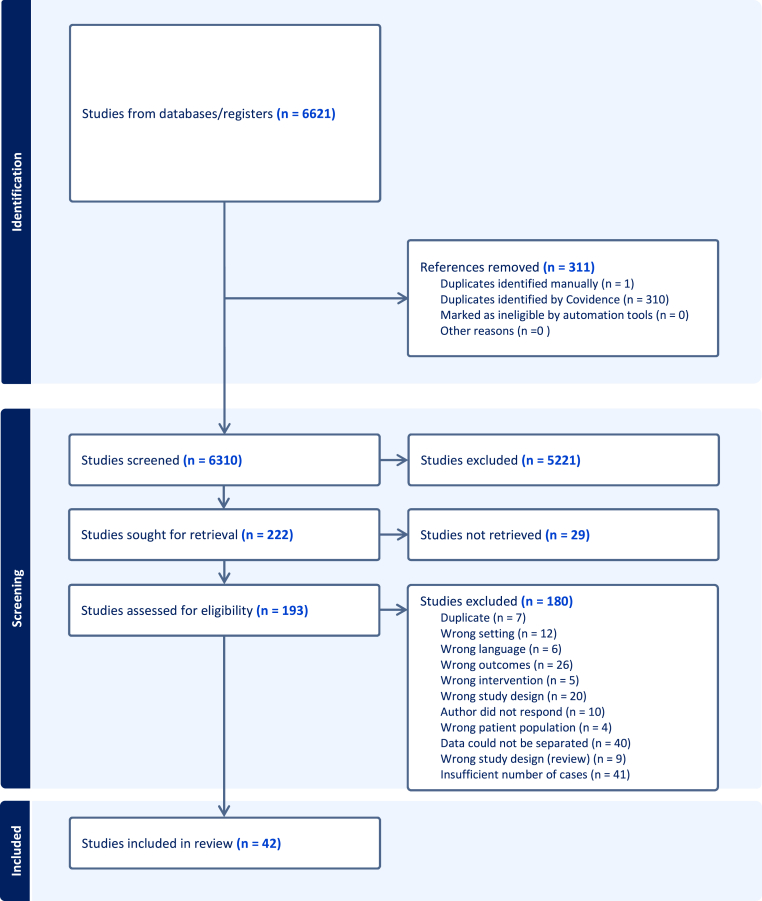
PRISMA flowchart.

In total, 6,621 studies were identified by the systematic literature search, of which 193 articles were eligible for full-text screening (Fig. 1). Twenty-nine studies could not be retrieved, and the most common reason for exclusion was insufficient cases. Therefore, this study included 42 studies.

### 3.2 Study characteristics

Six studies were prospective,^8^^,^^9^^,^^20^, ^21^, ^22^, ^23^ and the remaining 36 were retrospective^6^^,^^7^^,^^13^, ^14^, ^15^, ^16^, ^17^^,^^24^, ^25^, ^26^, ^27^, ^28^, ^29^, ^30^, ^31^, ^32^, ^33^, ^34^, ^35^, ^36^, ^37^, ^38^, ^39^, ^40^, ^41^, ^42^, ^43^, ^44^, ^45^, ^46^, ^47^, ^48^, ^49^, ^50^, ^51^, ^52^ (Table 1). The total number of included patients was 24,610, of which 2,418 had SSIs. The number of risk factors in each study varied from 1 to 26.

**Table 1:** Overview of studies.

Authors	Publication year	Country	Study design	Participants, *n*	Patients with infections	Minimum follow-up	Age^b^	Open/closed fractures	Patient Related Risk factors
Ashworth et al.^48^	2022	USA	Retrospective	151	36	90 days	44.8 ± 15.4	Both	9
Burrus et al.^24^	2015	USA	Retrospective	10213	1118	90 days	Uncertain	Both	1
Colman et al.^25^	2012	USA	Retrospective	309	24	12 months	47.8	Both	12
Doshi et al.^20^	2017	India	Prospective	768	23	12 months	40.1 ± 14	Both	6
Dubina et al.^26^	2017	USA	Retrospective	675	69	8 weeks	45.4	Uncertain	6
Duckworth et al.^13^	2016	UK	Retrospective	99	18	3 months	42 (16–86)	Both	5
Esposito et al.^17^	2019	USA	Retrospective	581	62	Uncertain	45 (35–55)	Both	8
Fonkoue et al.^52^	2023	Cameroon	Retrospective	105	33	12 months	37.9 ± 13	Both	13
Forni et al.^51^	2022	Brazil	Retrospective	44	11	Uncertain	48.5 ± 15.1	Both	12
Gaunder et al.^27^	2018	USA	Retrospective	102	16	1 month	>60	Both	10
Groznik et al.^28^	2019	Slovenia	Retrospective	86	20	Uncertain	69.3/66.8^a^	Both	8
Haase et al.^29^	2022	USA	Retrospective	244	34	3 months	50	Both	8
Henkelmann et al.^30^	2020	Germany	Retrospective	2106	94	Uncertain	50.2 ± 15.1	Both	12
Jenny et al.^33^	1994	France	Retrospective	359	20	Uncertain	Uncertain	Both	2
Kent et al.^31^	2015	UK	Retrospective	42	12	12 months	57.5/49.6^a^	Both	7
Kline et al.^32^	2009	USA	Retrospective	83	12	6 months	47	Both	1
Kugelman et al.^8^	2017	USA	Prospective	275	10	12 months	48.8 ± 14.63	Both	2
Li et al. (2018)^34^	2018	China	Retrospective	370	21	2 months	46.2	Both	16
Li et al. (2020)^21^	2020	China	Prospective	1108	25	12 months	45.6	Both	14
Lin et al.^35^	2013	USA	Retrospective	251	20	6 months	47.4/42.6^a^	Both	12
Ma et al.^36^	2018	China	Retrospective	676	17	Uncertain	44.4/46.9^a^	Both	18
Manon et al.^6^	2020	Belgium	Retrospective	168	13	Uncertain	45.6	Both	9
Messori et al.^49^	2023	Italy	Retrospective	103	12	12 months	49.9	Open	9
Metsemakers et al.^7^	2014	Belgium	Retrospective	480	21	18 months	Uncertain	Both	9
Molina et al.^37^	2015	USA	Retrospective	355	57	6 months	42.3 ± 14.2	Both	7
Momaya et al.^38^	2016	USA	Retrospectiv	532	59	19.5 months (Average)	47.76 ± 15.2	Both	13
Morris et al.^39^	2013	USA	Retrospective	302	43	14.1 months (Average)	45.7 ± 14.3 (19–76)	Both	9
Oladeji et al.^40^	2020	USA	Retrospective	276	46	12 months	43.8 ± 15.2	Both	1
Olesen et al.^41^	2015	Denmark	Retrospective	44	22	12 months	42 (16–71)	Open	4
Olson et al.^14^	2021	USA	Retrospective	161	43	12 months	46 ± 14	Open	4
Parkkinen et al.^42^	2016	Finland	Retrospective	170	34	12 months	55 (16–84)	Both	12
Ren et al.^43^	2015	China	Retrospective	519	12	12 months	>18	Both	10
Ruffolo et al.^16^	2015	USA	Retrospective	138	33	3 months	44.6 (16–78)	Both	6
Spitler et al.^44^	2020	USA	Retrospective	148	25	Uncertain	44	Both	10
Viberg et al.^15^	2016	Denmark	Retrospective	70	17	36 months	48 (15–85)	Both	6
Whiting et al.^22^	2019	USA, Kenya	Prospective	1061	113	4.9 months (Average)	35.5 ± 14	Both	6
Xie et al.^23^	2023	China	Prospective	417	30	12 months	45.6 ± 15.2	Both	12
Yeramosu et al.^45^	2022	USA	Retrospective	248	52	12 months	45.5/42.5^a^	Both	9
Ying et al.^22^	2023	China	Retrospective	351	51	30 days	44.0 (27.5–56.0)	Both	26
Yusof et al.^46^	2013	Malaysia	Retrospective	58	17	12 months	34.2/27.8^a^	Open	3
Zhu et al.^9^	2017	China	Prospective	235	12	12 months	45 (19–75)	Both	13
Zuelzer et al.^47^	2020	USA	Retrospective	127	11	6 weeks	37/41^a^	Open	7

a Mean for infection/no-infection.

b Presented as mean ± S.D. or range.

### 3.3 Risk of bias assessment

**Fig. 2:**
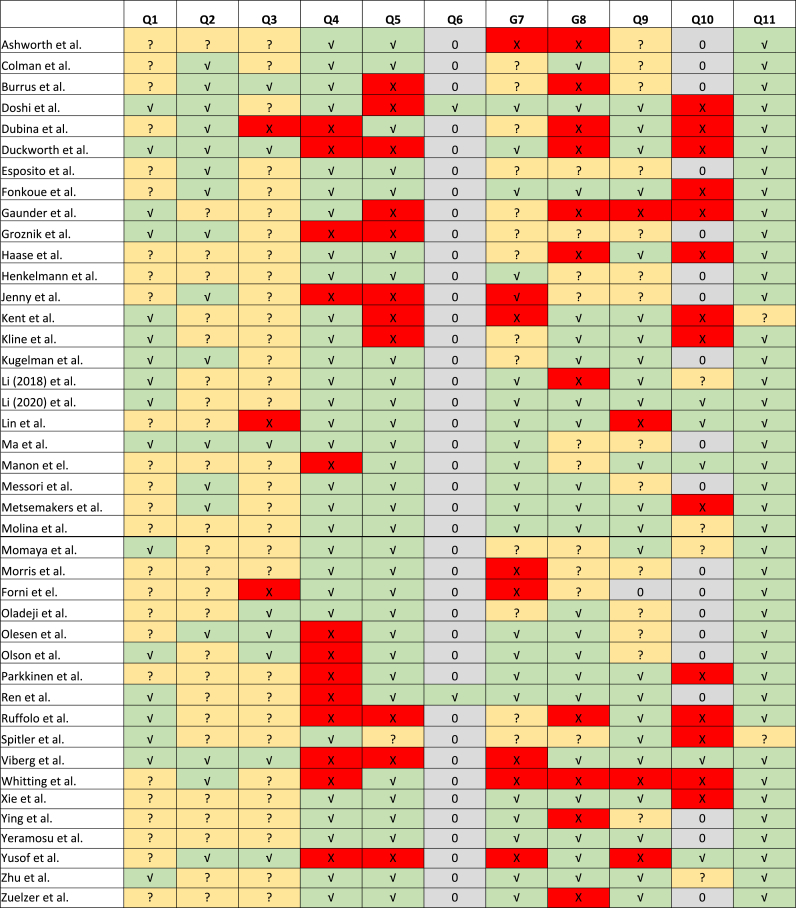
Risk of Bias Assessment Green (✓) is ‘Yes’, yellow (?) is ‘Unclear’, red (✕) is ‘No’ and grey (0) is ‘Not applicable’.

Study populations were generally similar and recruited from the same population (Fig. 2). Most studies identified confounding factors, and many performed multivariate regression analyses to adjust for confounders. Statistical analyses were performed accordingly, except for two studies.^31^^,^^44^

However, the definition and measurement validity and reliability for exposures were unclear. Only 21 of the 42 studies defined SSI clearly according to our criteria, increasing the risk of information bias. While 21 studies had >6 months of follow-up, nine had <3 months of follow-up, which could lead to an underestimation. Only a few studies described strategies for addressing incomplete follow-up, creating a substantial risk of selection bias due to differential loss to follow-up since these were all cohort studies.

### 3.4 Outcomes

**Fig. 3:**
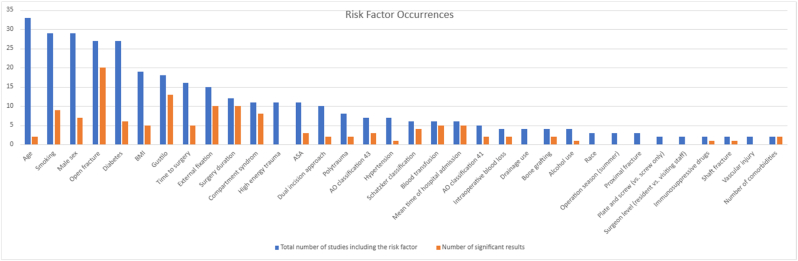
Risk Factor Occurrences Blue bar depicts total number of risk factor occurrences and orange bar depicts number of significant results.

Information on 43 potentially influencing risk factors was included from 42 studies (Fig. 3). Ten risk factors were only examined in one study and are therefore not shown in Fig. 3. Twenty risk factors were eligible for meta-analysis since they were investigated in ≥5 studies. The meta-analysis is summarised in Table 2, and the funnel and forest plots can be found in Appendix B. The remaining risk factors were examined in 2–4 studies and are listed in Table 3, including risk factors examined in only one study. Three studies were consistently excluded from meta-analyses because of missing data7,8,17.

**Table 2:** Overview of results from meta-analysis.

Risk factor	Studies included	OR (95% CI)	P-value
Sex (male)	29	1.6* (1.3–1.8)	<0.01
Diabetes	26	1.8 (0.95–3.3)	0.07
Smoking	28	1.8* (1.5–2.1)	<0.01
Open fracture	25	3.6* (2.5–5.1)	<0.01
Hypertension	7	1.2 (0.7–2.1)	0.49
Gustillo G3 vs G1+2	15	3.1* (2.1–4.6)	<0.01
ASA 3–4 vs 1-2	9	2.5* (1.5–4.1)	<0.01
Compartment	10	3.8* (2.4–6.0)	<0.01
Polytrauma	6	2.4* (1.5–4.0)	<0.01
High energy	11	1.5* (1.1–2.1)	0.02
External fixation	14	2.9* (2.3–3.8)	<0.01
Dual incision	7	2.1* (1.5–3.0)	<0.01
Duration of surgery	7	0.62* (0.43–0.82)	<0.01
Time to surgery	9	0.42 (−0.68-1.52)	0.45
Blood transfusion	6	3.8* (2.1–6.6)	<0.01

*Significant results with P-values <0.05.

**Table 3:** Risk factors divided based on occurrence of significant association with infection.

Significant result	Risk factors
**All studies**	Multiple comorbidities^13^^,^^29^
	IV drug use^30^
	Comminution^28^
	Pin-plate overlap^29^
	Nighttime surgery^30^
**50% of studies**	Intraoperative blood loss^9^^,^^21^^,^^34^^,^^36^
	Bone grafting^21^^,^^23^^,^^38^^,^^43^
	Immunosuppressive drugs^30^^,^^44^
	Fracture in shaft^22^^,^^52^
**<50% of studies**	Alcohol use^21^^,^^23^^,^^36^^,^^42^
**No studies**	Drainage use^9^^,^^34^^,^^36^^,^^43^
	Surgery during summer^9^^,^^34^^,^^36^
	Race^38^^,^^39^^,^^45^
	Proximal fracture location^22^^,^^41^^,^^52^
	Vascular injury^38^^,^^44^
	Surgeon level (resident vs. visiting staff)^9^^,^^34^
	Plate and screw vs. screw only^21^^,^^36^
	Distal fracture location^28^^,^^52^
	Tscherne classification^20^
	Public vs private hospital^20^
	Bone void filler^27^

A summary of fracture localization and risk factors can be seen in Appendix C. This table demonstrates that most data derive from tibia plateau fractures. We assumed that fracture localization did not influence the risk of infection. The table confirms this, as significant and not significant results are equally distributed across anatomical locations.

#### 3.4.1 Patient-related risk factors

There were seven patient-related risk factors; age, male sex, diabetes, American Association of Anesthesiologists (ASA) classification, obesity, smoking, and hypertension. Meta-analyses were possible for sex, diabetes, ASA classification, smoking, and hypertension, showing significant associations with male sex, ASA classification >2, and active smoking (Table 2). No studies clearly defined smoking based on the amount consumed currently or previously. Furthermore, no studies defined blood pressure limits for hypertension or defined diabetes.

The remaining risk factors are listed in Table 4, showing the number of studies that included them and found a significant result.

**Table 4:** Risk factors not eligible for meta-analysis.

Risk factor	Number of studies	Number of studies with significant results
**Age**	33	2^30^^,^^42^
**Obesity**	20	5^23^^,^^24^^,^^30^^,^^36^^,^^42^
**AO type 41B vs 41C**	5	2^30^^,^^42^
**AO type 43B vs 43C**	7	3^17^^,^^43^^,^^45^
**Schatzker**	6	4^21^^,^^26^^,^^36^^,^^38^

Age data were ineligible for meta-analysis since most studies provided median or mean values. The same applies to obesity, where results were listed as mean values or divided into subgroups based on body mass index.

Table 5 shows the excluded studies’ data.

**Table 5:** Data from studies not eligible for meta-analysis.

Risk factor	Odds ratio estimates (95% CI)
**Male sex**	2.1 (1.3–3.4)^17^^,^^b^
**Diabetes**	1.4 (0.7–3.1)^17^^,^^b^
	5.87 (0.21–165.96)^7^^,^^b^
**ASA classification**	4.1 (1.4–12.3)^42^^,^^a^
	1.20 (0.23–6.19)^b^
**Smoking**	1.74 (0.87–3.49)^7^^,^^b^^,^*
**Open fracture**	1.9 (1.2–3.0)^17^^,^^b^
	0.016 (0.001–0.269)^8^^,^^b^
	5.84 (0.23–145.50)^7^^,^^b^
**Gustilo-Anderson classification I + II vs III**	1.34 (0.53–3.40)^7^^,^^b^
**Compartment syndrome**	0.033 0.002–0.621)^8^^,^^b^
**Polytrauma**	0.27 (0.02–3.29)^7^^,^^b^
**Time to surgery**	1.1 (1.0–1.2)^42^
**External fixation**	2.0 (1.3–2.3)^17^
	27.0 (1.74–419.36)^7^
**Mean operative time**	1.3 (1.0–1.6)^42^^,^^b^
	1.78 (1.12–2.80)^25^
	2.72 (1.17–6.29)^34^^,^^b^

*Non-significant result p-values >0.05.

a Excluded due to different pooling of results.

b Excluded due to missing results.

#### 3.4.2 Fracture-related risk factors

There were seven fracture-related risk factors: open fracture, Gustilo–Anderson classification, compartment syndrome, polytrauma, high energy trauma, AO Foundation classification, and Schatzker classification. Meta-analyses were possible for open vs. closed fracture, Gustilo–Anderson classification type I + II vs. type III, compartment syndrome, polytrauma, and high-energy trauma, showing significant associations for all factors (Table 2).

While three studies stated that compartment syndrome was a clinical diagnosis and acutely treated with four-compartment fasciotomy,^25^^,^^26^^,^^42^ the remaining studies did not clarify the diagnosis further. Three studies defined polytrauma as having an Injury Severity Score >16^7,14,30^. One study defined polytrauma as fractures in multiple sites in the tibia.^28^ The remaining three studies quantified polytrauma by adding points when patients sustained trauma to different body parts.^9^^,^^34^^,^^35^ Finally, two studies did not define low vs. high-energy trauma.^44^ The remaining studies had very similar definitions, with low energy defined as falls from standing height^6^^,^^9^^,^^21^^,^^36^ and high energy as traffic incidents and falls from height.

The remaining risk factors are listed in Table 4, showing the number of studies that included them and found a significant finding.

A meta-analysis of AO type 41 and 43 fractures could not be performed since the numbers of patients in several articles’ infected groups were <10 after AO classification. Neither was a meta-analysis of Schatzker classification possible due to different grouping and missing data.^26^

#### 3.4.3 Surgery-related risk factors

There were five surgery-related risk factors: time to surgery, external fixation use, mean operative time, blood transfusions, and dual incision approach. Meta-analyses were possible for all factors, showing significant associations for external fixation use, blood transfusions, prolonged mean operative time, and dual incision approach (Table 2). The dual incision approach refers to operating through two separate incisions.

## 4 Discussion

In this systematic review, we conducted an extensive search to assess risk factors for postoperative tibial SSIs. To our knowledge, this is the first review to quantitively summarise existing data on risk factors for SSIs after ORIF for tibial fractures since our search found no existing reviews. We included 24,610 patients, of which 2,418 (9.8%) had SSIs, consistent with other similar studies.^4^^,^^5^^,^^53^^,^^54^

The systematic review included 42 studies, finding that SSIs were significantly associated with male sex, smoking, open fracture, Gustilo–Anderson score >2, blood transfusions, ASA score >2, compartment syndrome, high energy trauma, polytrauma, primary external fixation, dual incision approach, and prolonged surgery time.

Most included studies were retrospective cohort studies, often with significant selection and information bias risks. These studies have evident limitations since they depend directly on case documentation and lack traceability of further patient outcomes. Therefore, causality cannot be investigated and unambiguously established since any risk factors identified may be interactive and interrelated. For example, male patients could be more likely to sustain high-energy trauma or have higher alcohol consumption than female patients. Similarly, patients with polytrauma could be more likely to present with open fractures.

Despite these limitations, the current review has apparent advantages. We conducted a comprehensive literature search, including a larger sample size than similar reviews,^4^^,^^5^^,^^55^^,^^56^ enabling us to investigate numerous risk factors and collect data from up to 29 studies for a given risk factor. In case of missing data, authors were contacted in an attempt of collecting all existing data, this was however not possible to collect from all, contributing to risk of bias unpredictably.

Unlike other studies,^4^^,^^55^^,^^57^ diabetes was not a risk factor for SSIs in our analyses. While 26 studies examined diabetes mellitus as a risk factor for SSI, none defined diabetes or the presence of late complications or investigated the degree of glycemic control, increasing the risk of bias and possibly unpredictably affecting the results. If patients generally had well-controlled diabetes, this could explain the non-significant result to some extent. Furthermore, the mean age in population groups varied from 37 to 55 years in most studies and was 69 years in one study that only included patients aged >60 years.^27^ Therefore, the overall patient population was young and likely unaffected by late complications predisposing to SSIs.^57^^,^^58^ Finally, only 135 of 8.883 diabetic patients had SSIs. When events were divided into four groups for analysis, the number of diabetic patients with SSIs varied from zero to 16 across studies, with a mean of five. This low sample size reduced statistical power.

Compartment syndrome was the strongest predictor for SSIs (OR = 3.80), consistent with some studies^5^^,^^35^ and inconsistent with others.^16^^,^^59^^,^^60^ Ruffulo et al.^16^ found that a compartment syndrome diagnosis was not associated with increased SSI risk when pooling fasciotomy wounds closed primarily during or after definitive fixation. However, when examining only wounds closed secondarily, infection rates were significantly higher (OR = 7.5, *p* < 0.05). This finding may appear intuitively logical since open wounds are at greater risk of infection.^5^ As such, the presence of fasciotomy wounds may account for the increased risk of SSI. Zura et al.^60^ investigated how the timing of closing fasciotomy wounds affected SSI rates after ORIF for tibial plateau fractures, finding no significant association. Hak et al.^59^ showed that open fasciotomy wounds did not increase SSI risk after ORIF for tibial plateau fractures. However, both studies had low sample sizes and appreciable heterogeneity in study groups. Since little information exists on the optimal management of fasciotomy wounds concerning the subsequent definitive internal fixation,^59^ avoiding SSIs requires early compartment syndrome identification and implementing optimal wound care strategies.

Given the implications of SSIs, the importance of strategies for decreasing SSI risk is self-evident. While we cannot modify fracture-related risk factors, the high OR for SSIs with open fractures (OR = 3.6) and increasing Gustilo–Anderson score (OR = 3.1) emphasize the need for early intravenous antibiotic administration and adequate debridement. Further, a consequence of the current findings could be scheduled outpatient follow-ups including blood samples to detect SSIs sooner, especially when more risk factors are present. There is no evidence that patients benefit from postoperative continuation of prophylactic antibiotics^61^^,^^62^ but little data exist as to whether this also applies to patients with accumulating risk factors.

This leads to surgery-related risk factors; Blood transfusions were the second strongest predictor for SSIs (OR = 3.80). It is well established, that preoperative administration of prophylactic antibiotics prevents infection, but also that perioperative redosing is needed in case of excessive blood loss (i.e., >1,500 mL) to ensure adequate tissue and serum concentrations of the antimicrobial.^61^, ^62^, ^63^, ^64^ Included articles examining blood loss as a risk factor for SSI did not state, whether additional antibiotics were administered in case of excessive bleeding, one might speculate, that this was not the case.

The use of external fixation (OR = 2.9) and dual incision approach (OR = 2.1) were also predictors for SSI. While the operating physician decides on the use of external fixation and the number of incisions, this could also reflect a more complex and possibly open fracture with extensive soft tissue injury. Given that prolonged surgery time was also a risk factor for SSI, choosing a more experienced surgeon for high-risk patients could be wise to limit their surgery time. However, prolonged surgery time could also reflect more complex fractures.

Finally, attention must be given to preoperatively addressing potentially modifiable patient-related risk factors such as smoking (OR = 1.8) and ASA score (OR = 2.5), depending on the cause of the higher ASA scores.

## 5 Conclusions

This systematic review provides a basis for preoperatively assessing patient risk for developing a SSI, which could be used to initiate adjusted antibiotic regimes and more frequently postoperative controls. Furthermore, it indicates the risk factors future research should include when assessing for confounding factors.

This systematic review found that male sex, smoking, open fracture, a Gustilo–Anderson classification >2, an ASA classification >2, high energy trauma, blood transfusions, compartment syndrome, polytrauma, primary external fixation, dual incision approach, and prolonged surgery time were risk factors for SSI. However, the included studies were generally of poor quality and at risk of bias.

## Availability of data, code, and other material

If cited correctly, we can clarify whether the data are available upon reasonable request.

## Credit author statement

**Diana Niebuhr:** Validation, Investigation, Writing - Original Draft, Visualization. **Thomas Mattson:** Investigation. **Niels Martin Jensen:** Conceptualization, Methodology, Writing – Review & Editing. **Bjarke Viberg:** Conceptualization, Methodology, Writing – Review & Editing. **Signe Steenstrup Jensen:** Conceptualization, Methodology, Formal analysis, Writing – Review & Editing.

## Funding statement

This work did not receive any financial or non-financial support, and the authors made the decision to publish.

## Declaration of competing interest

The authors declare that they have no known competing financial interests or personal relationships that could have appeared to influence the work reported in this paper.

## References

[1] Thompson J.H. (2021).

[2] Trampuz A. (2006). Diagnosis and treatment of infections associated with fracture-fixation devices.

[3] Whitehouse J.D., Friedman N.D., Kirkland K.B., Richardson W.J., Sexton D.J. (2002). The impact of surgical-site infections following Orthopedic surgery at a community hospital and a university hospital adverse quality of life, excess length of stay, and extra cost. Infect Control Hosp Epidemiol.

[4] Shao J., Zhang H., Yin B., Li J., Zhu Y., Zhang Y. (2018). Risk factors for surgical site infection following operative treatment of ankle fractures: a systematic review and meta-analysis. Int J Surg.

[5] Shao J., Chang H., Zhu Y. (2017). Incidence and risk factors for surgical site infection after open reduction and internal fixation of tibial plateau fracture: a systematic review and meta-analysis. Int J Surg.

[6] Manon J., Detrembleur C., van de VeyVer S. (2020). Can Infect Predict Int Nailing Tibial Shaft Fract? Orig Stud.

[7] Metsemakers W.J., Handojo K., Reynders P., Sermon A., Vanderschot P., Nijs S. (2015). Individual risk factors for deep infection and compromised fracture healing after intramedullary nailing of tibial shaft fractures: a single centre experience of 480 patients. Injury.

[8] Kugelman D., Qatu A., Haglin J., Leucht P., Konda S., Egol K. (2017). Complications and unplanned outcomes following operative treatment of tibial plateau fractures. Injury.

[9] Zhu Y., Liu S., Zhang X., Chen W., Zhang Y. (2017). Incidence and risks for surgical site infection after adult tibial plateau fractures treated by ORIF: a prospective multicentre study. Int Wound J.

[10] Liberati A., Altman D.G., Tetzlaff J. (2009). The PRISMA statement for reporting systematic reviews and meta-analyses of studies that evaluate healthcare interventions: explanation and elaboration. BMJ.

[11] PROSPERO.

[12] Peduzzi P., Concato J., Kemper E., Holford T.R., Feinstein A.R. (1996). A simulation study of the number of events per variable in logistic regression analysis.

[13] Duckworth A.D., Jefferies J.G., Clement N.D., White T.O. (2016). Type C tibial pilon fractures. Bone Joint J Publ online.

[14] Olson J.J., Anand K., Esposito J.G. (2021). Complications and soft-tissue coverage after complete articular, open tibial plafond fractures. J Orthop Trauma.

[15] Viberg B., Kleven S., Hamborg-Petersen E., Skov O. (2016). Complications and functional outcome after fixation of distal tibia fractures with locking plate - a multicentre study. Injury.

[16] Ruffolo M.R., Gettys F.K., Montijo H.E., Seymour R.B., Karunakar M.A. (2014). http://www.jorthotrauma.com.

[17] Esposito J.G., van der Vliet Q.M.J., Heng M. (2020).

[18] Moola S Mztcaesksrcmqrmplkmpf. Chapter 7: Systematic Reviews of Etiology and Risk. JBI Manual for Evidence Synthesis.

[19] Tufanaru C., Munn Z., Stephenson M., Aromataris E. (2015). Fixed or random effects meta-analysis? Common methodological issues in systematic reviews of effectiveness. Int J Evid Base Healthc.

[20] Doshi P., Gopalan H., Sprague S., Pradhan C., Kulkarni S., Bhandari M. (2017). Incidence of infection following internal fixation of open and closed tibia fractures in India (INFINITI): a multi-centre observational cohort study. BMC Muscoskel Disord.

[21] Li J., Zhu Y., Zhao K. (2020). Incidence and risks for surgical site infection after closed tibial plateau fractures in adults treated by open reduction and internal fixation: a prospective study. J Orthop Surg Res.

[22] Whiting P.S., Galat D.D., Zirkle L.G., Shaw M.K., Galat J.D. (2019). Journal of Orthopaedic Trauma.

[23] Xie L., Liu G., Wang X. (2023). Development of a nomogram to predict surgical site infection after open reduction and internal fixation for closed pilon fracture: a prospective single-center study. J Orthop Surg Res.

[24] Burrus M.T., Werner B.C., Yarboro S.R. (2016). Obesity is associated with increased postoperative complications after operative management of tibial shaft fractures. Injury.

[25] Colman M., Wright A., Gruen G., Siska P., Pape H.C., Tarkin I. (2013). Prolonged operative time increases infection rate in tibial plateau fractures. Injury.

[26] Dubina A.G., Paryavi E., Manson T.T., Allmon C., O'Toole R v (2017). Surgical site infection in tibial plateau fractures with ipsilateral compartment syndrome. Injury.

[27] Gaunder C.L., Zhao Z., Henderson C., McKinney B.R., Stahel P.F., Zelle B.A. (2019). Wound complications after open reduction and internal fixation of tibial plateau fractures in the elderly: a multicentre study. Int Orthop.

[28] Groznik M., Cimerman M., Lusa L., Gorenjec N.R., Ihan A. (2019). Increased perioperative C-reactive protein and decreased postoperative albumin is associated with acute posttraumatic osteomyelitis in patients with high-energy tibial fractures. Injury.

[29] Haase L.R., Haase D.R., Moon T.J. (2022). Is pin-plate overlap in tibial plateau fractures associated with increased infection rates?. Injury.

[30] Henkelmann R., Frosch K.H., Mende M. (2021). Risk factors for deep surgical site infection in patients with operatively treated tibial plateau fractures: a retrospective multicenter study. J Orthop Trauma.

[31] Kent M., Mumith A., McEwan J., Hancock N. (2015). The service impact of failed locking plate fixation of distal tibial fractures: a service and financial evaluation at a major trauma centre. Eur J Orthop Surg Traumatol.

[32] Kline A.J., Gruen G.S., Pape H.C., Tarkin I.S., Irrgang J.J., Wukich D.K. (2009). Early complications following the operative treatment of pilon fractures with and without diabetes. Foot Ankle Int.

[33] Jenny J.Y., Jenny G., Kempf I. (1994). Infection after reamed intramedullary nailing of lower limb fractures: a review of 1,464 cases over 15 years. Acta Orthop.

[34] Li J., Zhu Y., Liu B., Dong T., Chen W., Zhang Y. (2018). Incidence and risk factors for surgical site infection following open reduction and internal fixation of adult tibial plateau fractures. Int Orthop.

[35] Lin S., Mauffrey C., Hammerberg E.M., Stahel P.F., Hak D.J. (2014). Surgical site infection after open reduction and internal fixation of tibial plateau fractures. Eur J Orthop Surg Traumatol.

[36] Ma Q., Aierxiding A., Wang G., Wang C., Yu L., Shen Z. (2018). Incidence and risk factors for deep surgical site infection after open reduction and internal fixation of closed tibial plateau fractures in adults. Int Wound J.

[37] Molina C.S., Stinner D.J., Fras A.R., Evans J.M. (2015). Risk factors of deep infection in operatively treated pilon fractures (AO/OTA: 43). J Orthop.

[38] Momaya A.M., Hlavacek J., Etier B. (2016). Risk factors for infection after operative fixation of Tibial plateau fractures. Injury.

[39] Morris B.J., Unger R.Z., Archer K.R., Mathis S.L., Perdue A.M., Obremskey W.T. (2013). Risk factors of infection after ORIF of bicondylar tibial plateau fractures. http://www.jorthotrauma.com.

[40] Oladeji L.O., Platt B., Crist B.D. (2021). Diabetic pilon factures: are they as bad as we think?. J Orthop Trauma.

[41] Olesen U.K., Juul R., Bonde C.T. (2015). A review of forty five open tibial fractures covered with free flaps. Analysis of complications, microbiology and prognostic factors. Int Orthop.

[42] Parkkinen M., Madanat R., Lindahl J., Mäkinen T.J. (2016). Risk factors for deep infection following plate fixation of proximal tibial fractures. J Bone Joint Surg- American.

[43] Ren T., Ding L., Xue F., He Z., Xiao H. (2015). Risk factors for surgical site infection of pilon fractures. Clinics.

[44] Spitler C.A., Hulick R.M., Weldy J., Howell K., Bergin P.F., Graves M.L. (2020). What are the risk factors for deep infection in OTA/AO 43C pilon fractures?. J Orthop Trauma.

[45] Yeramosu T., Satpathy J., Perdue P.W. (2022). Risk factors for infection and subsequent adverse clinical results in the setting of operatively treated pilon fractures. J Orthop Trauma.

[46] Yusof NM, Khalid KA, Hafiz Zulkifly A, Zakaria Z, Azril M, Amin M, et al. Factors Associated with the Outcome of Open Tibial Fractures. www.mjms.usm.my. PMC3957352 24643115

[47] Zuelzer D.A., Hayes C.B., Hautala G.S. (2021). Early antibiotic administration is associated with a reduced infection risk when combined with primary wound closure in patients with open tibia fractures. Clin Orthop Relat Res.

[48] Ashworth T.J., Alvarez P.M., Laux J.P., Ganga S., Ostrum R.F. (2022). http://www.c-orthopaedicpractice.com.

[49] Messori M., Touloupakis G., Gilli A. (2023). The risk of infection in open distal tibial fracture: the DANGER score. Eur J Orthop Surg Traumatol.

[50] Ying H., Guo B.W., Wu H.J., Zhu R.P., Liu W.C., Zhong H.F. (2023). Using multiple indicators to predict the risk of surgical site infection after ORIF of tibia fractures: a machine learning based study. Front Cell Infect Microbiol.

[51] Nogueira Forni J.E., Tardivo Fraga S.E., Jalikj W. (2022). Risk factors for infection in patients undergoing osteosynthesis for tibial plateau fracture in a university hospital. Cureus. Publ online April.

[52] Fonkoue L., Tissingh E.K., Muluem O.K. (2023). Predictive factors for fracture-related infection in open tibial fractures in a Sub-Saharan African setting. Injury.

[53] de Carvalho R.L.R., Campos C.C., Franco LM. de C., Rocha A. de M., Ercole F.F. (2017). Incidence and risk factors for surgical site infection in general surgeries. Rev Lat Am Enfermagem.

[54] Mu Y., Edwards J.R., Horan T.C., Berrios-Torres S.I., Fridkin S.K. (2011). Improving risk-adjusted measures of surgical site infection for the national healthcare safely network. Infect Control Hosp Epidemiol.

[55] Gortler H., Rusyn J., Godbout C., Chahal J., Schemitsch E.H., Nauth A. (2018). Diabetes and healing outcomes in lower extremity fractures: a systematic review. Injury.

[56] Wang TaoGJunfeiLYubin (2023). Predictors of acute compartment syndrome in patients with tibial fractures: a meta-analysis. Int Orthop.

[57] Dane K. (2011). Wukich MAJDMRDCRBJJIPPA. Outcomes of ankle fractures in patients with uncomplicated VersusComplicated diabetes. Foot Ankle Int.

[58] Jones K.B., Maiers-Yelden K.A., Marsh J.L. (2005). Ankle fractures in patients with diabetes mellitus.

[59] Hak Djmmlmmgdr Do (2010). Influence of prior fasciotomy on infection after open reduction and internal fixation of tibial plateau fractures. Injury.

[60] Zura R. Adams S. Jeray K. Obremskey W. Stinett S. Olson S. Timing of definitive fixation of severe tibial plateau fractures with compartment syndrome does not have an effect on the rate of infection. J Trauma Inj Infect Crit Care. 2010;69(6):1523–1526. 10.1097/TA.0b013e3181d40403 20495494

[61] Noble D.W.G.I. (2012). Antibiotics for surgical patients: the faster the better?. Lancet Infect Dis.

[62] Bratzler D.W., Dellinger E.P., Olsen K.M. (2013). Clinical practice guidelines for antimicrobial prophylaxis in surgery. Surg Infect.

[63] Zelenitsky S.A., Ariano R.E., Harding G.K.M., Silverman R.E. (2002). Antibiotic pharmacodynamics in surgical prophylaxis: an association between intraoperative antibiotic concentrations and efficacy. Antimicrob Agents Chemother.

[64] Swoboda SM, Merz C, Kostuik J, Trentler B, Lipsett PA. Does Intraoperative Blood Loss Affect Antibiotic Serum and Tissue Concentrations?. 10.1001/archsurg.1996.01430230047009 8911256

