# Supplementary material for: Risk factors for tibial infections following osteosynthesis – a systematic review and meta-analysis
Source: J Clin Orthop Trauma. 2024 Feb 23;50:102376. doi: 10.1016/j.jcot.2024.102376 (PMC10909754; doi:10.1016/j.jcot.2024.102376)
Supplement: Multimedia component 1 [file mmc1.docx]

# – Appendix A

# Search databases and synonyms

## Cochrane Library

| #1 | MeSH descriptor: [Fracture Fixation, Internal] explode all trees | 1550 |
| --- | --- | --- |
| #2 | fract* fixat* | 5471 |
| #3 | osteosynthes* | 1386 |
| #4 | MeSH descriptor: [Open Fracture Reduction] explode all trees | 45 |
| #5 | "open reduction" | 1199 |
| #6 | MeSH descriptor: [Closed Fracture Reduction] explode all trees | 20 |
| #7 | "Closed reduction" | 612 |
| #8 | Bone fixa* | 4267 |
| #9 | MeSH descriptor: [Fracture Fixation] explode all trees | 1960 |
| #10 | "Fracture reduction" | 707 |
| #11 | Skeletal fixa* | 271 |
| #12 | MeSH descriptor: [Fracture Fixation, Intramedullary] explode all trees | 386 |
| #13 | intramedullary nailing | 781 |
| #14 | Intramedul* fract* fixa* | 863 |
| #15 | Osteosynthesis intramedullary | 197 |
| #16 | Intramedullary nail* | 1116 |
| #17 | Marrow nail* | 55 |
| #18 | Medullary nail* | 68 |
| #19 | MeSH descriptor: [Bone Screws] explode all trees | 883 |
| #20 | Bone screw* | 2529 |
| #21 | skelet* screw* | 245 |
| #22 | fract* screw* | 2056 |
| #23 | Cannulated screw* | 191 |
| #24 | Compression screw* | 375 |
| #25 | Biodegradable screw* | 71 |
| #26 | Lock* screw* | 357 |
| #27 | Fixati* Screw* | 2277 |
| #28 | MeSH descriptor: [Bone Nails] explode all trees | 454 |
| #29 | bone nail* | 1221 |
| #30 | Nail* fixa* | 1181 |
| #31 | skelet* nail* | 119 |
| #32 | fract* nail* | 1755 |
| #33 | bone pin* | 2109 |
| #34 | Pin fixa* | 390 |
| #35 | skelet* pin* | 501 |
| #36 | fract* pin* | 2015 |
| #37 | Rod fixa* | 123 |
| #38 | Bone rod* | 1366 |
| #39 | skelet* rod* | 505 |
| #40 | fract* rod* | 1342 |
| #41 | Surgical wire | 667 |
| #42 | Intraosseous wire* | 11 |
| #43 | Wire* fixat* | 615 |
| #44 | MeSH descriptor: [Bone Wires] explode all trees | 191 |
| #45 | bone wire* | 635 |
| #46 | skelet* wire* | 162 |
| #47 | fract* wire* | 820 |
| #48 | Kirschner wire* | 260 |
| #49 | MeSH descriptor: [Bone Plates] explode all trees | 677 |
| #50 | bone plate* | 6340 |
| #51 | Compression plate* | 691 |
| #52 | Plate fixa* | 1623 |
| #53 | fract* plate* | 4005 |
| #54 | skelet* plate* | 643 |
| #55 | MeSH descriptor: [Suture Anchors] explode all trees | 79 |
| #56 | Suture anchor* | 446 |
| #57 | Bone Anchor | 155 |
| #58 | Anchor fixa* | 110 |
| #59 | cerclage | 486 |
| #60 | tension band* | 452 |
| #61 | #1 OR #2 OR #3 OR #4 OR #5 OR #6 OR #7 OR #8 OR #9 OR #10 OR #11 OR #12 OR #13 OR #14 OR #15 OR #16 OR #17 OR #18 OR #19 OR #20 OR #21 OR #22 OR #23 OR #24 OR #25 OR #26 OR #27 OR #28 OR #29 OR #30 OR #31 OR #32 OR #33 OR #34 OR #35 OR #36 OR #37 OR #38 OR #39 OR #40 #41 OR #42 OR #43 OR #44 OR #45 OR #46 OR #47 OR #48 OR #49 OR #50 OR #51 OR #52 OR #53 OR #54 OR #55 OR #56 OR #57 OR #58 OR #59 OR #60 | 22279 |
| #62 | MeSH descriptor: [Risk Factors] explode all trees | 26359 |
| #63 | Risk factor* | 114851 |
| #64 | "Population at risk" | 847 |
| #65 | MeSH descriptor: [Risk] explode all trees | 39648 |
| #66 | Risk* | 283437 |
| #67 | MeSH descriptor: [Comorbidity] explode all trees | 3852 |
| #68 | comorbid* | 30470 |
| #69 | Epidemiological data | 7490 |
| #70 | MeSH descriptor: [Morbidity] explode all trees | 15966 |
| #71 | Morbid* | 53837 |
| #72 | Multidiseas* | 34 |
| #73 | Multi-diseas* | 22 |
| #74 | MeSH descriptor: [Multiple Chronic Conditions] explode all trees | 48 |
| #75 | Multiple chronic conditions | 4114 |
| #76 | Multimorbid* | 729 |
| #77 | Multi-morbid* | 156 |
| #78 | Population Characteristic* | 22655 |
| #79 | Predict* | 113429 |
| #80 | MeSH descriptor: [Epidemiology] explode all trees | 42 |
| #81 | epidemiolog* | 80064 |
| #82 | "smoking cessation" | 11773 |
| #83 | MeSH descriptor: [Smoking Cessation] explode all trees | 4476 |
| #84 | MeSH descriptor: [Smoke] explode all trees | 440 |
| #85 | smoking | 37316 |
| #86 | MeSH descriptor: [Cigarette Smoking] explode all trees | 167 |
| #87 | cigarette smoking | 6603 |
| #88 | MeSH descriptor: [Smoking] explode all trees | 6543 |
| #89 | "cigar smoking" | 12 |
| #90 | tobacco smok* | 15374 |
| #91 | MeSH descriptor: [Obesity] 4 tree(s) exploded | 15907 |
| #92 | Obesi* | 46935 |
| #93 | Adiposi* | 3312 |
| #94 | Excess body weight | 2275 |
| #95 | MeSH descriptor: [Overnutrition] explode all trees | 19208 |
| #96 | Overnutrition | 226 |
| #97 | Overweight | 19975 |
| #98 | MeSH descriptor: [Body Weight] explode all trees | 31561 |
| #99 | Body weight | 76696 |
| #100 | MeSH descriptor: [Diabetes Mellitus] explode all trees | 35722 |
| #101 | Diabet* | 113860 |
| #102 | MeSH descriptor: [Alcohols] explode all trees | 39244 |
| #103 | Alcoholism | 8699 |
| #104 | Alcohol dependence | 5813 |
| #105 | Alcohol* | 38833 |
| #106 | MeSH descriptor: [Alcoholism] explode all trees | 3972 |
| #107 | Alcohol abuse | 5996 |
| #108 | Alcohol addiction | 3373 |
| #109 | nonsteroid antiinflammatory agent | 1623 |
| #110 | Anti-Inflammatory Agents, Non-Steroidal | 7544 |
| #111 | MeSH descriptor: [Anti-Inflammatory Agents] explode all trees | 13911 |
| #112 | antiinflammatory agent | 4990 |
| #113 | anti inflammat* drug* | 29624 |
| #114 | nonsteroid antiinflammatory agent | 1623 |
| #115 | #62 OR #63 OR #64 OR #65 OR #66 OR #67 OR #68 OR #69 OR #70 OR #71 OR #72 OR #73 OR #74 OR #75 OR #76 OR #77 OR #78 OR #79 OR #80 OR #81 OR #82 OR #83 OR #84 OR #85 OR #86 OR #87 OR #88 OR #89 OR #90 OR #91 OR #92 OR #93 OR #94 OR #95 OR #96 OR #97 OR #98 OR #99 OR #100 OR #101 OR #102 OR #103 OR #104 OR #105 OR #106 OR #107 OR #108 OR #109 OR #110 OR #111 OR #112 OR #113 OR #114 | 654009 |
| #116 | MeSH descriptor: [Bone Diseases, Infectious] explode all trees | 424 |
| #117 | MeSH descriptor: [Sepsis] explode all trees | 4986 |
| #118 | (sepsis):ti,ab,kw (Word variations have been searched) | 12507 |
| #119 | MeSH descriptor: [Soft Tissue Infections] explode all trees | 146 |
| #120 | MeSH descriptor: [Suppuration] explode all trees | 1270 |
| #121 | MeSH descriptor: [Abscess] explode all trees | 669 |
| #122 | MeSH descriptor: [Wound Infection] explode all trees | 4167 |
| #123 | MeSH descriptor: [Equipment Contamination] explode all trees | 393 |
| #124 | MeSH descriptor: [Infections] explode all trees | 82755 |
| #125 | MeSH descriptor: [Osteomyelitis] explode all trees | 155 |
| #126 | (((infection*) OR (infect* NEXT bone*) OR (fracture* NEXT infect*) OR (sepsis) OR (blood NEXT poisoning*) OR (soft NEXT tissue* NEXT infect*) OR (suppuration*) OR (pus*) OR (absces*) OR (co*infect*) OR (coinfect*) OR (equipment* NEXT contaminat*) OR (osteomyelit*))):ti,ab,kw (Word variations have been searched) | 146103 |
| #127 | #116 OR #117 OR #118 OR #119 OR #120 OR #121 OR #122 OR #123 OR #124 OR #125 OR #126 | 177347 |
| #128 | MeSH descriptor: [Tibia] explode all trees | 670 |
| #129 | (((tibia*) OR (shin*) OR (bone* NEXT flute*) OR (cnemis*) OR (crus) OR (low* NEXT leg*) OR (shank*))):ti,ab,kw (Word variations have been searched) | 9563 |
| #130 | #128 OR #129 | 9563 |
| #131 | #61 AND #115 AND #127 AND #130 | 10 |

## Embase/Medline

| Search # | Annotations |
| --- | --- |
| 1 | Risk factor*.mp. or exp risk factor/ |
| 2 | Population* at risk*.mp. |
| 3 | Comorbid*.mp. or exp comorbidity/ |
| 4 | Epidemiological data.mp. or exp epidemiological data/ |
| 5 | Morbid*.mp. or exp morbidity/ |
| 6 | Multidiseas*.mp. |
| 7 | Multi-diseas*.mp. |
| 8 | Multiple chronic condition*.mp. or exp multiple chronic conditions/ |
| 9 | Multimorbid*.mp. |
| 10 | Multi-morbid*.mp. |
| 11 | Population* Characteristic*.mp. |
| 12 | Predictor*.mp. |
| 13 | exp epidemiology/ or Epidemiology.mp. |
| 14 | exp smoking/ or exp cigarette smoking/ or Smoking.mp. or exp smoking habit/ or exp cigar smoking/ |
| 15 | smok*.mp. |
| 16 | tobac*.mp. |
| 17 | exp obesity/ or Obesity.mp. |
| 18 | Obesi*.mp. |
| 19 | Adipos*.mp. |
| 20 | Excess body weight.mp. |
| 21 | Overnutrition.mp. or exp overnutrition/ |
| 22 | Overweight.mp. |
| 23 | Body weight.mp. or exp body weight/ |
| 24 | Diabetes mellitus.mp. or exp diabetes mellitus/ |
| 25 | Diabet*.mp. |
| 26 | Alcoholism.mp. or exp alcoholism/ |
| 27 | Alcohol*.mp. |
| 28 | exp alcohol abuse/ |
| 29 | Anti-Inflammatory Agents, Non-Steroidal.mp. or exp nonsteroid antiinflammatory agent/ |
| 30 | antiinflammatory agent.mp. or exp antiinflammatory agent/ |
| 31 | anti inflammat* drug*.mp. |
| 32 | nonsteroid antiinflammatory agent.mp. or exp nonsteroid antiinflammatory agent/ |
| 33 | nsaid*.mp. |
| 34 | ibupro*.mp. |
| 35 | ipren*.mp. |
| 36 | 1 or 2 or 3 or 4 or 5 or 6 or 7 or 8 or 9 or 10 or 11 or 12 or 13 or 14 or 15 or 16 or 17 or 18 or 19 or 20 or 21 or 22 or 23 or 24 or 25 or 26 or 27 or 28 or 29 or 30 or 31 or 32 or 33 or 34 or 35 |
| 37 | exp compression osteosynthesis/ or exp osteosynthesis/ or exp osteosynthesis material/ |
| 38 | osteosynthes*.mp. [mp=title, abstract, heading word, drug trade name, original title, device manufacturer, drug manufacturer, device trade name, keyword heading word, floating subheading word, candidate term word] |
| 39 | exp fracture fixation/ |
| 40 | fracture* fixat*.mp. [mp=title, abstract, heading word, drug trade name, original title, device manufacturer, drug manufacturer, device trade name, keyword heading word, floating subheading word, candidate term word] |
| 41 | exp "open reduction (procedure)"/ |
| 42 | open reduct*.mp. [mp=title, abstract, heading word, drug trade name, original title, device manufacturer, drug manufacturer, device trade name, keyword heading word, floating subheading word, candidate term word] |
| 43 | exp "closed reduction (procedure)"/ |
| 44 | close* reduct*.mp. [mp=title, abstract, heading word, drug trade name, original title, device manufacturer, drug manufacturer, device trade name, keyword heading word, floating subheading word, candidate term word] |
| 45 | bone* fixat*.mp. [mp=title, abstract, heading word, drug trade name, original title, device manufacturer, drug manufacturer, device trade name, keyword heading word, floating subheading word, candidate term word] |
| 46 | exp fracture reduction/ |
| 47 | fracture* reduct*.mp. [mp=title, abstract, heading word, drug trade name, original title, device manufacturer, drug manufacturer, device trade name, keyword heading word, floating subheading word, candidate term word] |
| 48 | exp external fixator/ |
| 49 | extern* fixat*.mp. [mp=title, abstract, heading word, drug trade name, original title, device manufacturer, drug manufacturer, device trade name, keyword heading word, floating subheading word, candidate term word] |
| 50 | skelet* fixat*.mp. [mp=title, abstract, heading word, drug trade name, original title, device manufacturer, drug manufacturer, device trade name, keyword heading word, floating subheading word, candidate term word] |
| 51 | exp intramedullary nailing/ |
| 52 | intramedul* nail*.mp. [mp=title, abstract, heading word, drug trade name, original title, device manufacturer, drug manufacturer, device trade name, keyword heading word, floating subheading word, candidate term word] |
| 53 | marrow* nail*.mp. [mp=title, abstract, heading word, drug trade name, original title, device manufacturer, drug manufacturer, device trade name, keyword heading word, floating subheading word, candidate term word] |
| 54 | medul* nail*.mp. [mp=title, abstract, heading word, drug trade name, original title, device manufacturer, drug manufacturer, device trade name, keyword heading word, floating subheading word, candidate term word] |
| 55 | exp bone screw/ |
| 56 | bone* screw*.mp. [mp=title, abstract, heading word, drug trade name, original title, device manufacturer, drug manufacturer, device trade name, keyword heading word, floating subheading word, candidate term word] |
| 57 | skelet* screw*.mp. [mp=title, abstract, heading word, drug trade name, original title, device manufacturer, drug manufacturer, device trade name, keyword heading word, floating subheading word, candidate term word] |
| 58 | fracture* screw*.mp. [mp=title, abstract, heading word, drug trade name, original title, device manufacturer, drug manufacturer, device trade name, keyword heading word, floating subheading word, candidate term word] |
| 59 | exp cannulated screw/ |
| 60 | cannulat* screw*.mp. [mp=title, abstract, heading word, drug trade name, original title, device manufacturer, drug manufacturer, device trade name, keyword heading word, floating subheading word, candidate term word] |
| 61 | exp compression screw/ |
| 62 | compres* screw*.mp. [mp=title, abstract, heading word, drug trade name, original title, device manufacturer, drug manufacturer, device trade name, keyword heading word, floating subheading word, candidate term word] |
| 63 | exp biodegradable implant/ or exp biodegradable screw/ |
| 64 | biodegrad* screw*.mp. [mp=title, abstract, heading word, drug trade name, original title, device manufacturer, drug manufacturer, device trade name, keyword heading word, floating subheading word, candidate term word] |
| 65 | biodegrad* implant*.mp. [mp=title, abstract, heading word, drug trade name, original title, device manufacturer, drug manufacturer, device trade name, keyword heading word, floating subheading word, candidate term word] |
| 66 | exp locking screw/ |
| 67 | Lock* screw*.mp. [mp=title, abstract, heading word, drug trade name, original title, device manufacturer, drug manufacturer, device trade name, keyword heading word, floating subheading word, candidate term word] |
| 68 | fixat* screw*.mp. [mp=title, abstract, heading word, drug trade name, original title, device manufacturer, drug manufacturer, device trade name, keyword heading word, floating subheading word, candidate term word] |
| 69 | exp bone nail/ |
| 70 | bone* nail*.mp. [mp=title, abstract, heading word, drug trade name, original title, device manufacturer, drug manufacturer, device trade name, keyword heading word, floating subheading word, candidate term word] |
| 71 | fixat* nail*.mp. [mp=title, abstract, heading word, drug trade name, original title, device manufacturer, drug manufacturer, device trade name, keyword heading word, floating subheading word, candidate term word] |
| 72 | exp intramedullary nail/ |
| 73 | skelet* nail*.mp. [mp=title, abstract, heading word, drug trade name, original title, device manufacturer, drug manufacturer, device trade name, keyword heading word, floating subheading word, candidate term word] |
| 74 | fract* nail*.mp. [mp=title, abstract, heading word, drug trade name, original title, device manufacturer, drug manufacturer, device trade name, keyword heading word, floating subheading word, candidate term word] |
| 75 | exp bone pin/ |
| 76 | exp fracture external fixation/ |
| 77 | fract* extern* fixat*.mp. [mp=title, abstract, heading word, drug trade name, original title, device manufacturer, drug manufacturer, device trade name, keyword heading word, floating subheading word, candidate term word] |
| 78 | bone* pin*.mp. [mp=title, abstract, heading word, drug trade name, original title, device manufacturer, drug manufacturer, device trade name, keyword heading word, floating subheading word, candidate term word] |
| 79 | pin* fixat*.mp. [mp=title, abstract, heading word, drug trade name, original title, device manufacturer, drug manufacturer, device trade name, keyword heading word, floating subheading word, candidate term word] |
| 80 | skelet* pin*.mp. [mp=title, abstract, heading word, drug trade name, original title, device manufacturer, drug manufacturer, device trade name, keyword heading word, floating subheading word, candidate term word] |
| 81 | fract* pin*.mp. [mp=title, abstract, heading word, drug trade name, original title, device manufacturer, drug manufacturer, device trade name, keyword heading word, floating subheading word, candidate term word] |
| 82 | rod* fixat*.mp. [mp=title, abstract, heading word, drug trade name, original title, device manufacturer, drug manufacturer, device trade name, keyword heading word, floating subheading word, candidate term word] |
| 83 | bone* rod*.mp. [mp=title, abstract, heading word, drug trade name, original title, device manufacturer, drug manufacturer, device trade name, keyword heading word, floating subheading word, candidate term word] |
| 84 | skelet* rod*.mp. [mp=title, abstract, heading word, drug trade name, original title, device manufacturer, drug manufacturer, device trade name, keyword heading word, floating subheading word, candidate term word] |
| 85 | fract* rod*.mp. [mp=title, abstract, heading word, drug trade name, original title, device manufacturer, drug manufacturer, device trade name, keyword heading word, floating subheading word, candidate term word] |
| 86 | exp surgical wire/ or exp wire fixation/ |
| 87 | surg* wire*.mp. [mp=title, abstract, heading word, drug trade name, original title, device manufacturer, drug manufacturer, device trade name, keyword heading word, floating subheading word, candidate term word] |
| 88 | wire* fixat*.mp. [mp=title, abstract, heading word, drug trade name, original title, device manufacturer, drug manufacturer, device trade name, keyword heading word, floating subheading word, candidate term word] |
| 89 | intraoss* wire*.mp. [mp=title, abstract, heading word, drug trade name, original title, device manufacturer, drug manufacturer, device trade name, keyword heading word, floating subheading word, candidate term word] |
| 90 | exp Kirschner wire/ or exp bone wire/ |
| 91 | exp bone wire/ |
| 92 | bone* wire*.mp. [mp=title, abstract, heading word, drug trade name, original title, device manufacturer, drug manufacturer, device trade name, keyword heading word, floating subheading word, candidate term word] |
| 93 | skelet* wire*.mp. [mp=title, abstract, heading word, drug trade name, original title, device manufacturer, drug manufacturer, device trade name, keyword heading word, floating subheading word, candidate term word] |
| 94 | fract* wire*.mp. [mp=title, abstract, heading word, drug trade name, original title, device manufacturer, drug manufacturer, device trade name, keyword heading word, floating subheading word, candidate term word] |
| 95 | kirscher* wire*.mp. [mp=title, abstract, heading word, drug trade name, original title, device manufacturer, drug manufacturer, device trade name, keyword heading word, floating subheading word, candidate term word] |
| 96 | K* wire*.mp. [mp=title, abstract, heading word, drug trade name, original title, device manufacturer, drug manufacturer, device trade name, keyword heading word, floating subheading word, candidate term word] |
| 97 | exp bone plate/ |
| 98 | bone* plate*.mp. [mp=title, abstract, heading word, drug trade name, original title, device manufacturer, drug manufacturer, device trade name, keyword heading word, floating subheading word, candidate term word] |
| 99 | exp compression plate/ |
| 100 | compres* plate*.mp. [mp=title, abstract, heading word, drug trade name, original title, device manufacturer, drug manufacturer, device trade name, keyword heading word, floating subheading word, candidate term word] |
| 101 | exp plate fixation/ |
| 102 | plate* fixat*.mp. [mp=title, abstract, heading word, drug trade name, original title, device manufacturer, drug manufacturer, device trade name, keyword heading word, floating subheading word, candidate term word] |
| 103 | fract* plate*.mp. [mp=title, abstract, heading word, drug trade name, original title, device manufacturer, drug manufacturer, device trade name, keyword heading word, floating subheading word, candidate term word] |
| 104 | skelet* plate*.mp. [mp=title, abstract, heading word, drug trade name, original title, device manufacturer, drug manufacturer, device trade name, keyword heading word, floating subheading word, candidate term word] |
| 105 | exp suture anchor/ |
| 106 | sutur* anchor*.mp. [mp=title, abstract, heading word, drug trade name, original title, device manufacturer, drug manufacturer, device trade name, keyword heading word, floating subheading word, candidate term word] |
| 107 | bone* anchor*.mp. [mp=title, abstract, heading word, drug trade name, original title, device manufacturer, drug manufacturer, device trade name, keyword heading word, floating subheading word, candidate term word] |
| 108 | anchor* fixat*.mp. [mp=title, abstract, heading word, drug trade name, original title, device manufacturer, drug manufacturer, device trade name, keyword heading word, floating subheading word, candidate term word] |
| 109 | exp cerclage/ |
| 110 | cerclage*.mp. [mp=title, abstract, heading word, drug trade name, original title, device manufacturer, drug manufacturer, device trade name, keyword heading word, floating subheading word, candidate term word] |
| 111 | tension* band*.mp. [mp=title, abstract, heading word, drug trade name, original title, device manufacturer, drug manufacturer, device trade name, keyword heading word, floating subheading word, candidate term word] |
| 112 | 37 or 38 or 39 or 40 or 41 or 42 or 43 or 44 or 45 or 46 or 47 or 48 or 49 or 50 or 51 or 52 or 53 or 54 or 55 or 56 or 57 or 58 or 59 or 60 or 61 or 62 or 63 or 64 or 65 or 66 or 67 or 68 or 69 or 70 or 71 or 72 or 73 or 74 or 75 or 76 or 77 or 78 or 79 or 80 or 81 or 82 or 83 or 84 or 85 or 86 or 87 or 88 or 89 or 90 or 91 or 92 or 93 or 94 or 95 or 96 or 97 or 98 or 99 or 100 or 101 or 102 or 103 or 104 or 105 or 106 or 107 or 108 or 109 or 110 or 111 |
| 113 | exp infection/ or exp device infection/ or exp cross infection/ |
| 114 | exp wound infection/ |
| 115 | exp mixed infection/ or exp superinfection/ or exp secondary infection/ or exp bacterial infection/ |
| 116 | exp bone infection/ |
| 117 | exp soft tissue infection/ |
| 118 | exp surgical infection/ |
| 119 | exp postoperative complication/ or exp infection complication/ |
| 120 | exp contamination/ or exp bacterium contamination/ or exp medical device contamination/ or exp microbial contamination/ |
| 121 | exp medical device contamination/ or exp equipment/ |
| 122 | exp osteomyelitis/ or exp chronic osteomyelitis/ |
| 123 | exp bone disease/ |
| 124 | exp pus/ |
| 125 | exp abscess/ or exp skin abscess/ or exp abscess drainage/ |
| 126 | exp sepsis/ |
| 127 | exp suppuration/ |
| 128 | (infect* or infect* bone* or fracture* infect* or sepsis or blood poisoning* or soft tissue* infection* or suppuration* or pus* or abscess* or co*infection* or coinfect* or equipment* contamination* or osteomyelit*).mp. [mp=title, abstract, heading word, drug trade name, original title, device manufacturer, drug manufacturer, device trade name, keyword heading word, floating subheading word, candidate term word] |
| 129 | 113 or 114 or 115 or 116 or 117 or 118 or 119 or 120 or 121 or 122 or 123 or 124 or 125 or 126 or 127 or 128 |
| 130 | exp proximal tibia/ or exp tibia/ or exp tibia shaft fracture/ or exp distal tibia fracture/ or exp proximal tibia fracture/ or exp tibia fracture/ or exp distal tibia/ |
| 131 | tibia.mp. [mp=title, abstract, heading word, drug trade name, original title, device manufacturer, drug manufacturer, device trade name, keyword heading word, floating subheading word, candidate term word] |
| 132 | tibial.mp. [mp=title, abstract, heading word, drug trade name, original title, device manufacturer, drug manufacturer, device trade name, keyword heading word, floating subheading word, candidate term word] |
| 133 | shin*.mp. [mp=title, abstract, heading word, drug trade name, original title, device manufacturer, drug manufacturer, device trade name, keyword heading word, floating subheading word, candidate term word] |
| 134 | bone* flute*.mp. [mp=title, abstract, heading word, drug trade name, original title, device manufacturer, drug manufacturer, device trade name, keyword heading word, floating subheading word, candidate term word] |
| 135 | cnemis.mp. [mp=title, abstract, heading word, drug trade name, original title, device manufacturer, drug manufacturer, device trade name, keyword heading word, floating subheading word, candidate term word] |
| 136 | crus.mp. [mp=title, abstract, heading word, drug trade name, original title, device manufacturer, drug manufacturer, device trade name, keyword heading word, floating subheading word, candidate term word] |
| 137 | low* leg*.mp. [mp=title, abstract, heading word, drug trade name, original title, device manufacturer, drug manufacturer, device trade name, keyword heading word, floating subheading word, candidate term word] |
| 138 | shank*.mp. [mp=title, abstract, heading word, drug trade name, original title, device manufacturer, drug manufacturer, device trade name, keyword heading word, floating subheading word, candidate term word] |
| 139 | 130 or 131 or 132 or 133 or 134 or 135 or 136 or 137 or 138 |
| 140 | 36 and 112 and 129 and 139 |

## Scopus

( ( ( TITLE-ABS-KEY ( "risk factor*" OR "population* at risk*" OR "comorbid*" OR "epidemiological data" OR morbid* OR multidiseas* OR "multi* diseas*" OR "multiple chronic condition*" OR multimorbid* OR "multi-morbid*" OR "population characteristic*" OR predictor* OR epidemiolo* OR "smoking cessation" OR smok* OR cigarette* OR "smoking habit*" OR "cigar smoking" OR tobacco* OR obesi* OR adiposi* OR "excess body weight" OR overnutriti* OR overweight* OR "body weight" OR diabet* OR alcohol* OR "anti inflammatory" OR "anti-inflammatory" OR nonsteroid* OR nsaid OR ibupro* OR ipren* ) ) ) ) AND ( ( ( TITLE-ABS-KEY ( osteosynthes* OR "fracture* fixat*" OR "open reduc*" OR "closed reduc" * OR "bone* fixa*" OR "frac* reduct*" OR "extern* fixa*" OR "skelet* fixat*" OR "intramedul* nail*" OR "marrow* nail*" OR "medul* nail*" OR "bone* screw*" OR "skelet* screw*" OR "fracture* screw*" OR "fracture* screw*" OR "cannulat* screw*" OR "compress* screw*" OR "biodegrad* screw*" OR "biodegrad* implant*" OR "lock* screw*" OR "fixat* screw*" OR "bone* nail*" OR "fixat* nail*" OR "skelet* nail*" OR "fract* nail*" OR "bone* pin*" OR "pin* fixat*" OR "skelet* pin*" OR "fract* pin*" OR "rod* fixat*" OR "bone* rod*" OR "skelet* rod*" OR "fract* rod*" OR "surg* wire*" OR "intraoss* wire*" OR "kirschn* wire*" OR "bone* wire*" OR "skelet* wire*" OR "fract* wire*" OR "k* wire*" OR "bone* plate*" OR "compress* plate*" OR "plate* fixat*" OR "fract* plate*" OR "skelet* plate*" OR "sutur* anchor*" OR "bone* anchor*" OR "anchor* fixat*" OR cerclage* OR "tension* band*" ) ) ) ) AND ( ( ( TITLE-ABS-KEY ( infection* OR "postoperativ* complicatio*" OR contamination* OR osteomyelit* OR "bone* disease*" OR pus* OR absces* OR sepsis* OR suppuration* ) ) ) ) AND ( ( ( TITLE-ABS-KEY ( tibia* OR tibial* OR shin* OR "bone* flute*" OR cnemis* OR crus* OR "low* leg*" OR shank* ) ) ) )

No limitations were used.
