# Supplementary material for: Risk factors for tibial infections following osteosynthesis – a systematic review and meta-analysis
Source: J Clin Orthop Trauma. 2024 Feb 23;50:102376. doi: 10.1016/j.jcot.2024.102376 (PMC10909754; doi:10.1016/j.jcot.2024.102376)

# – Appendix B

Forrest- and funnelplots

Diabetes


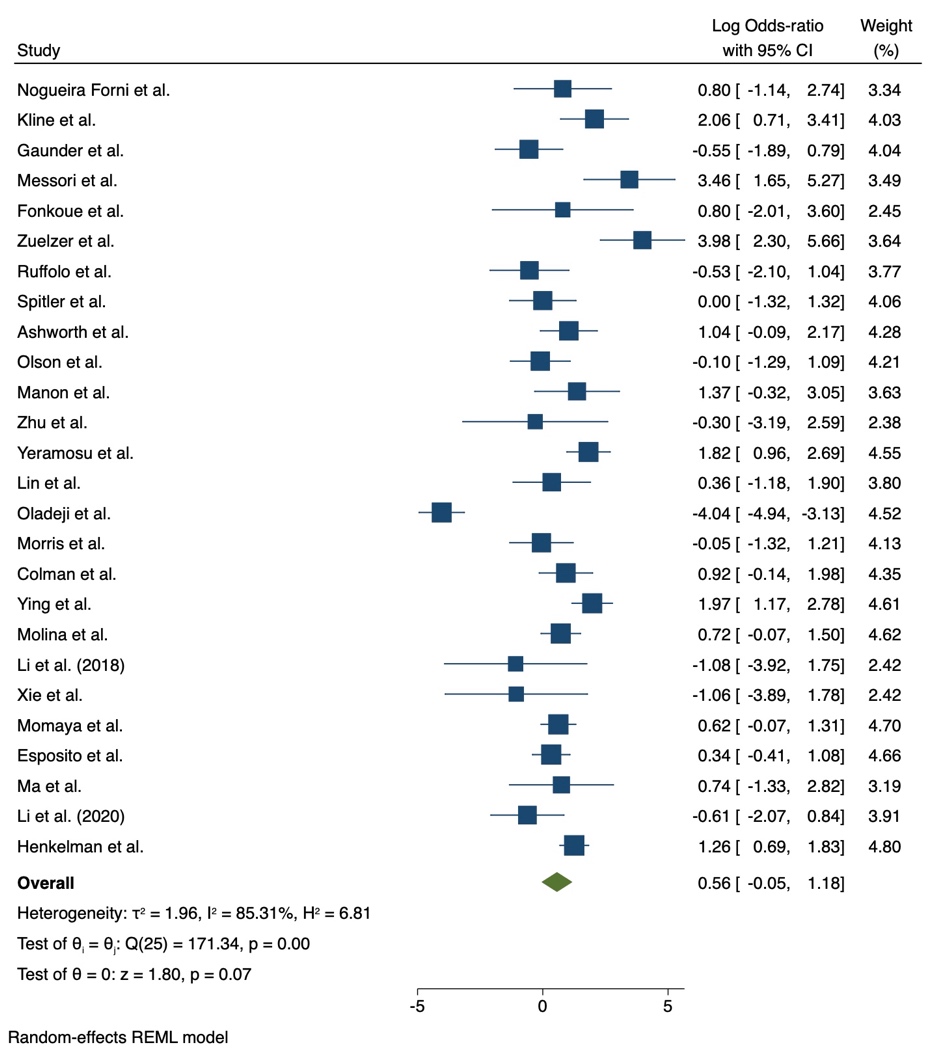


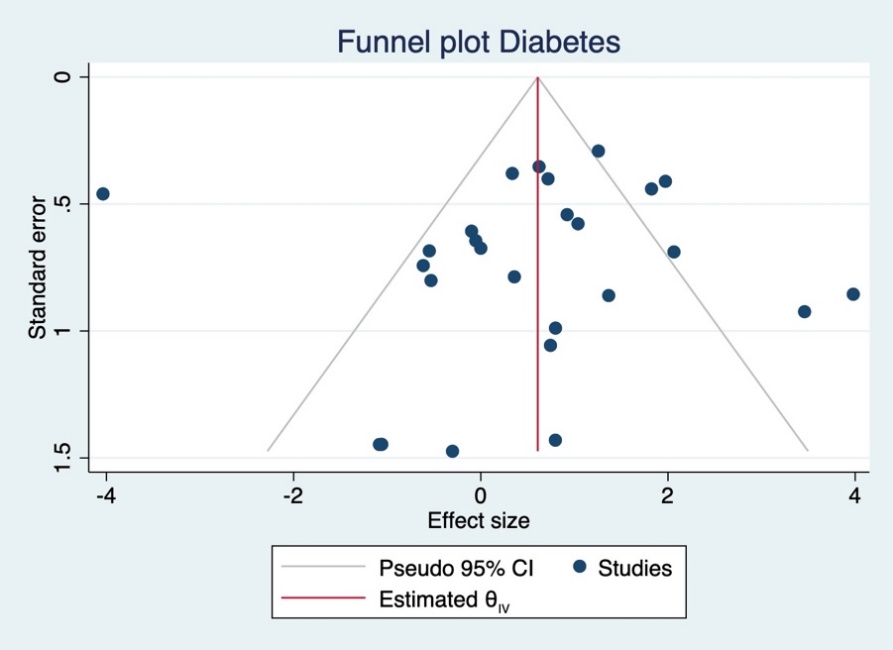


Sex


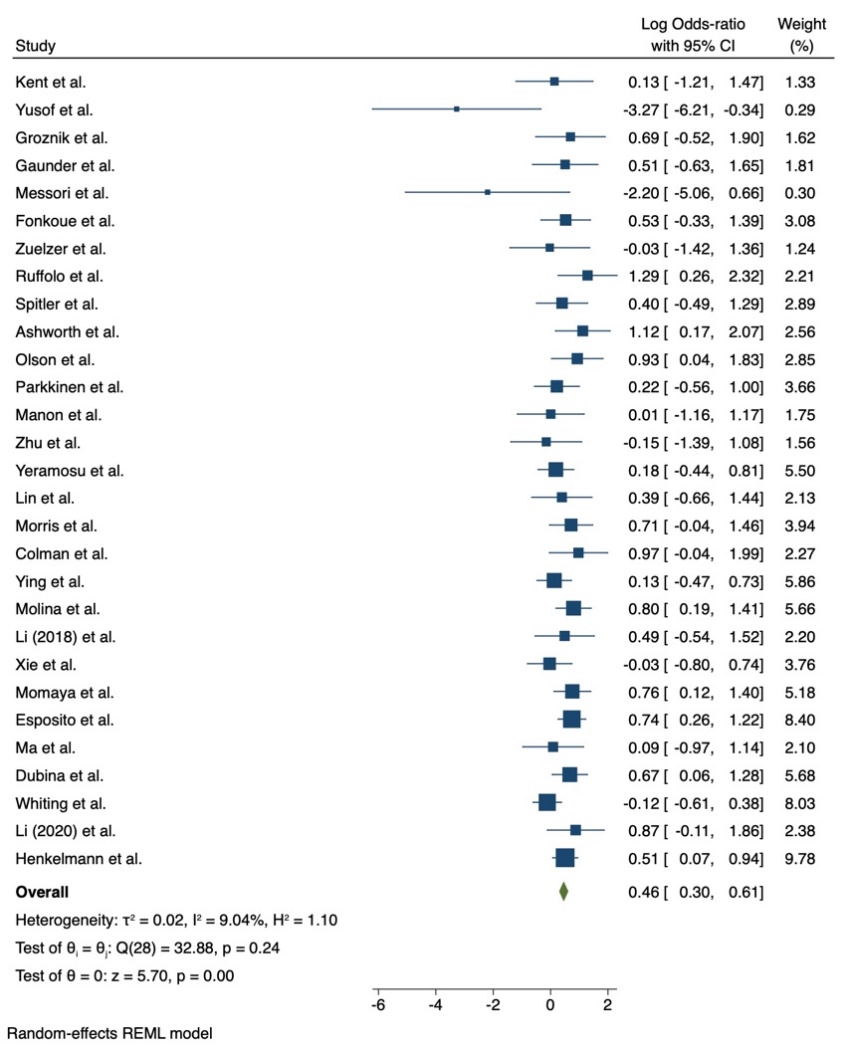


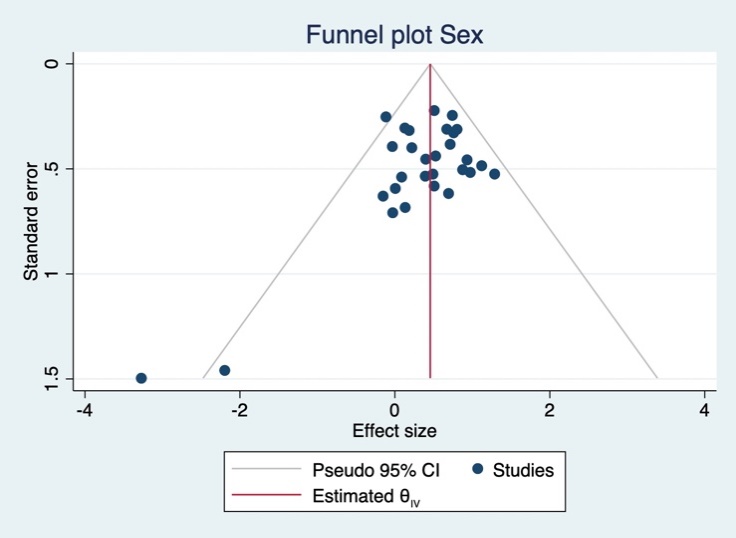


Smoking


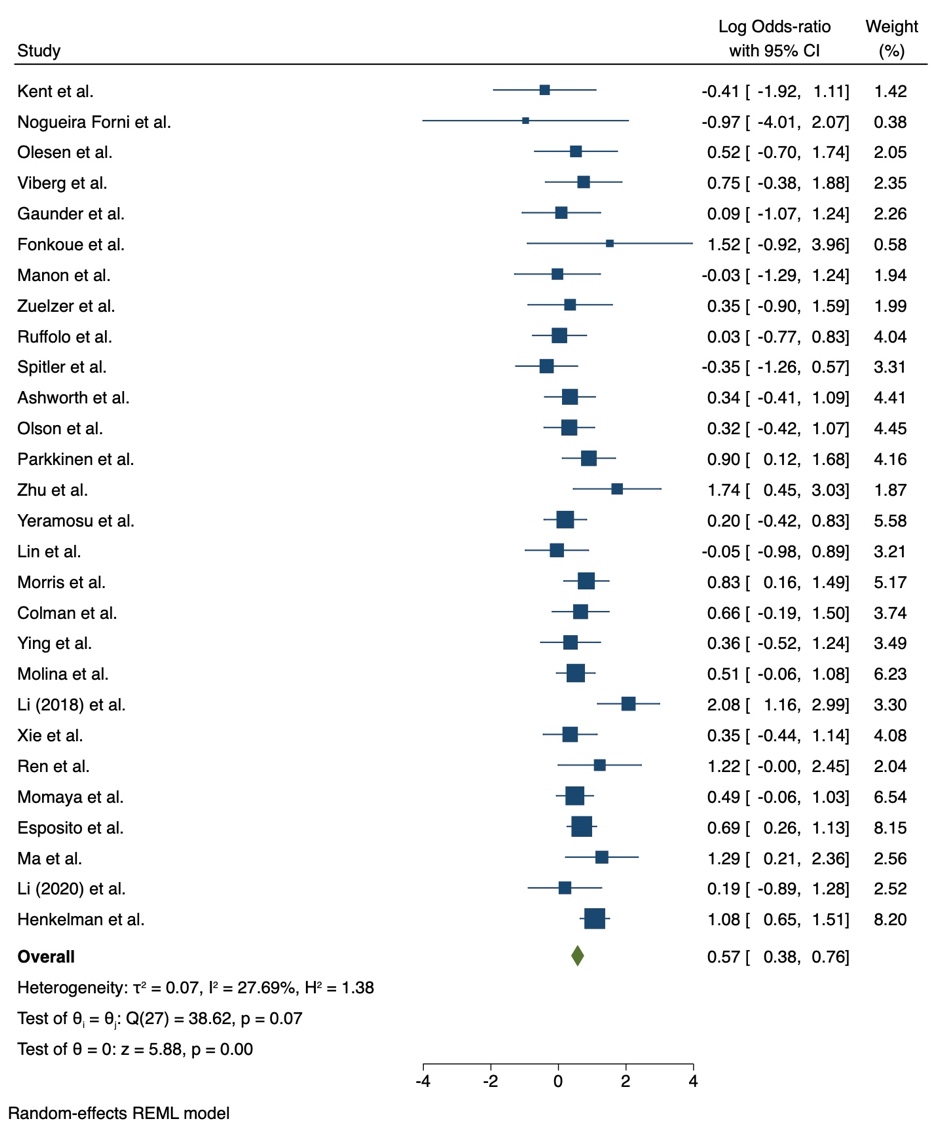


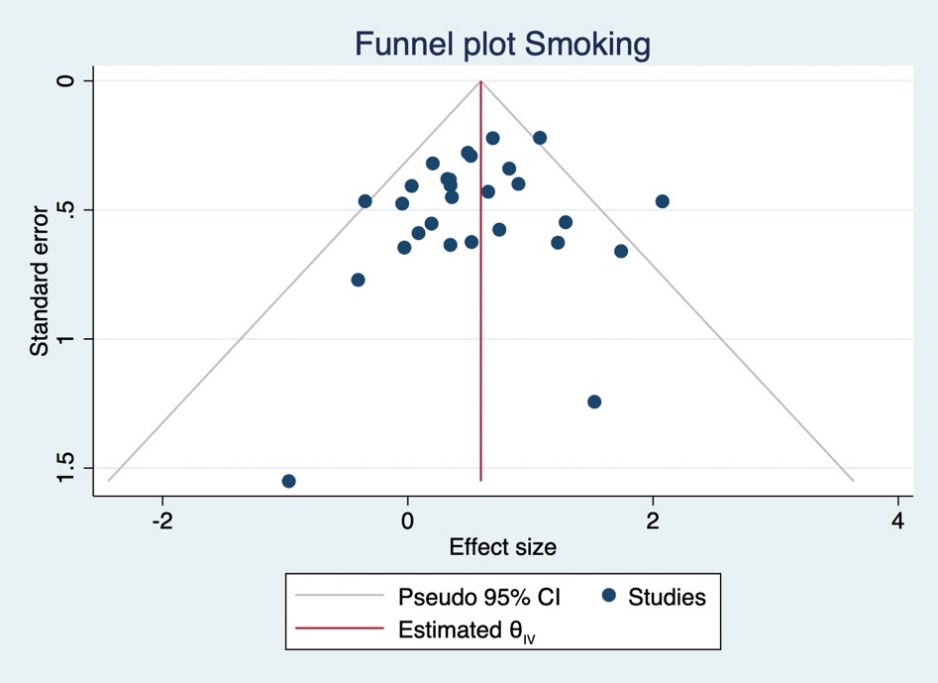


Open fracture


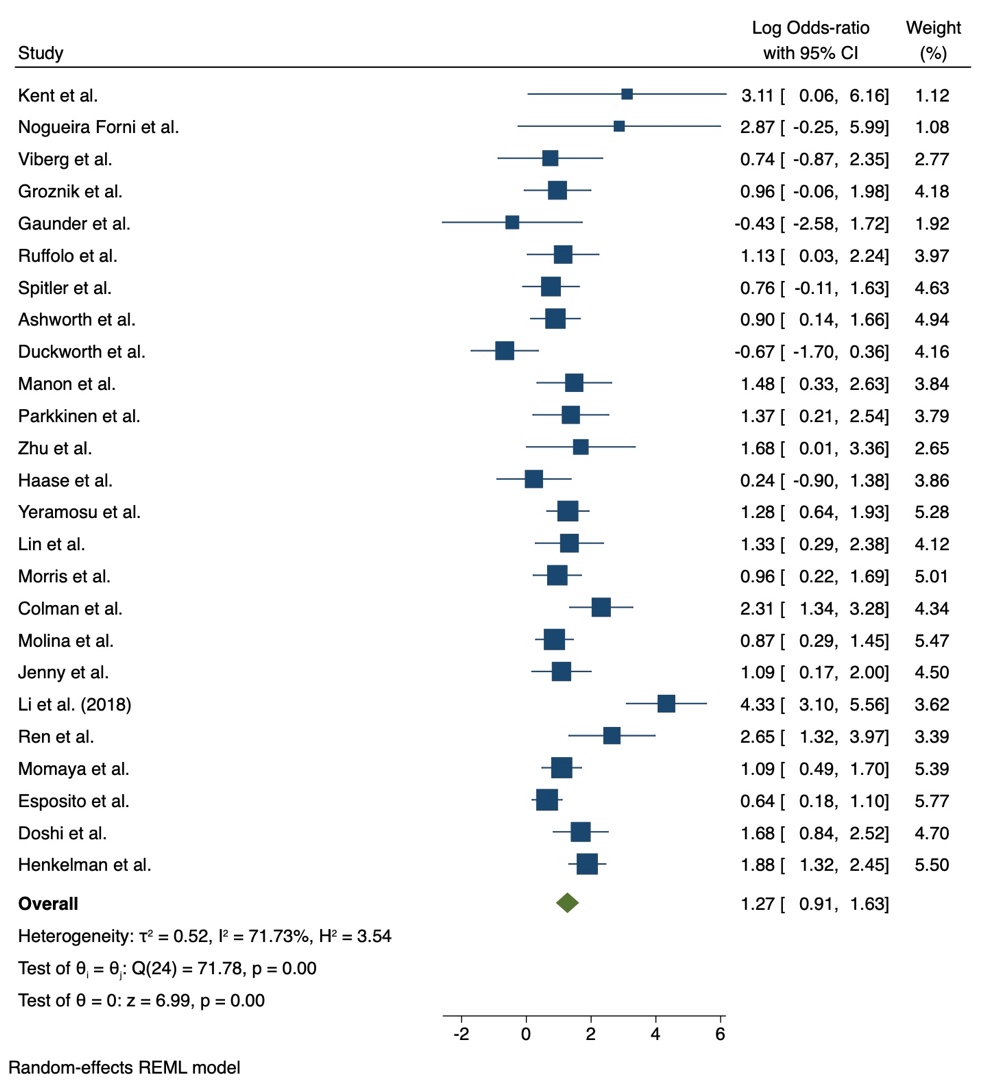


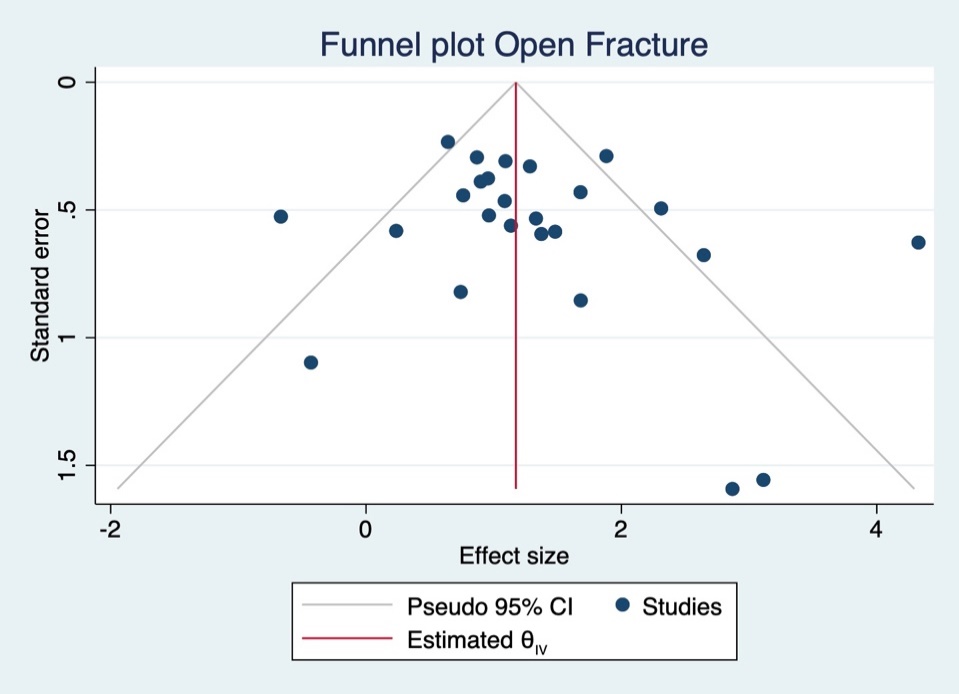


Hypertension


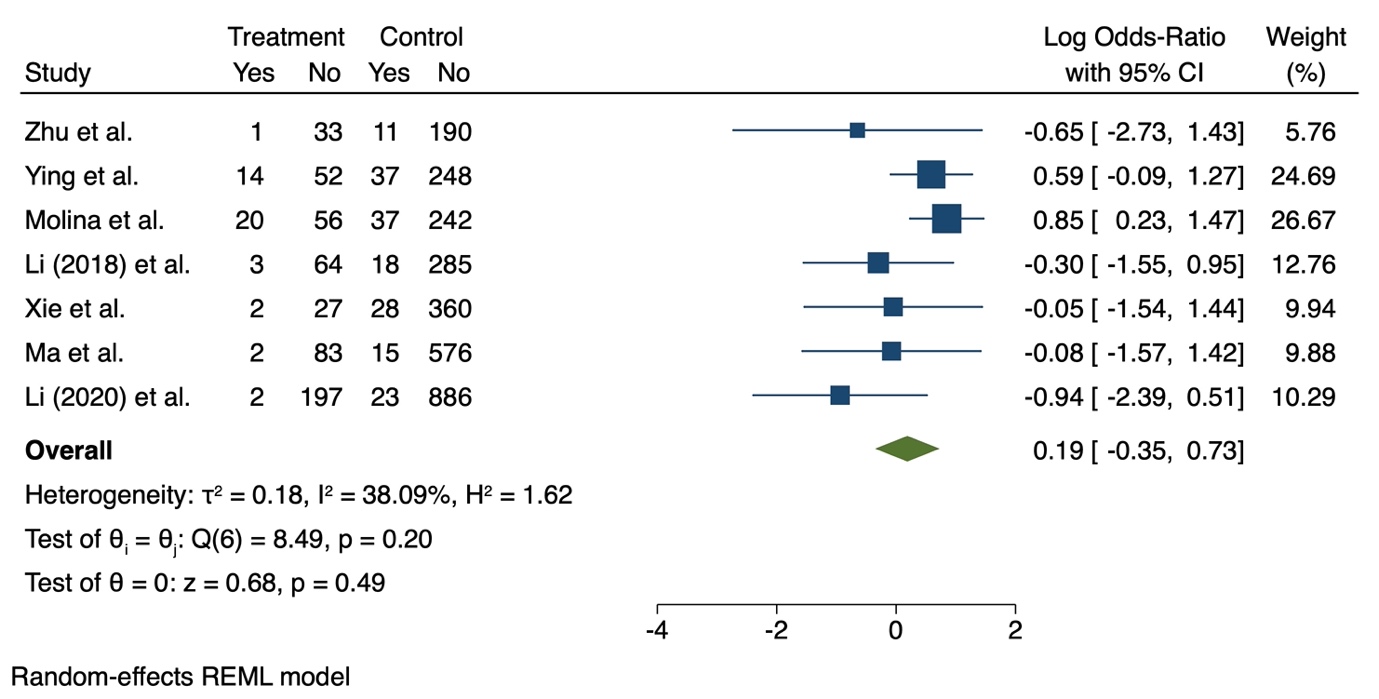


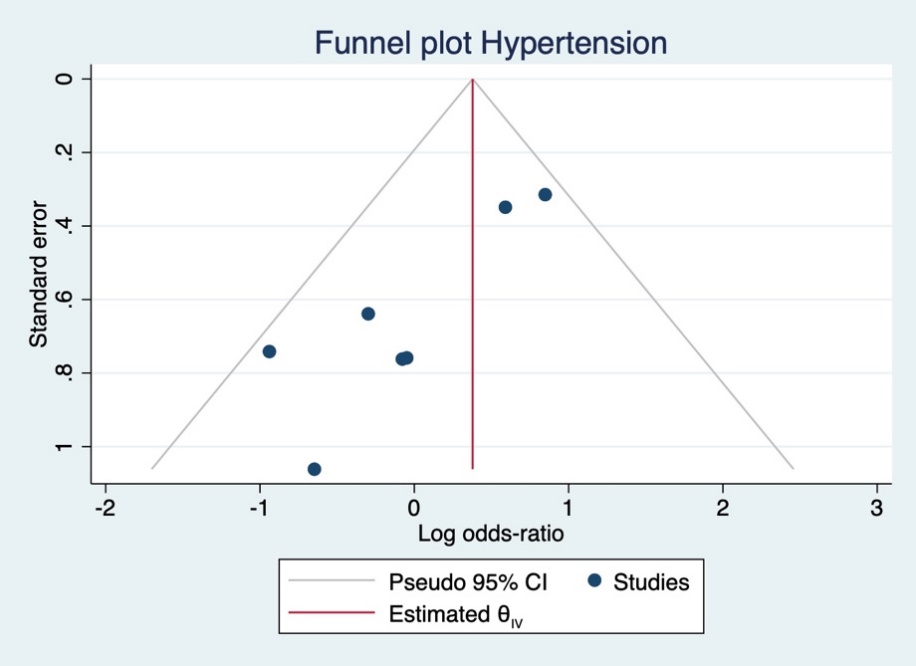


Gustilo-Anderson classification 3 vs. 1+2


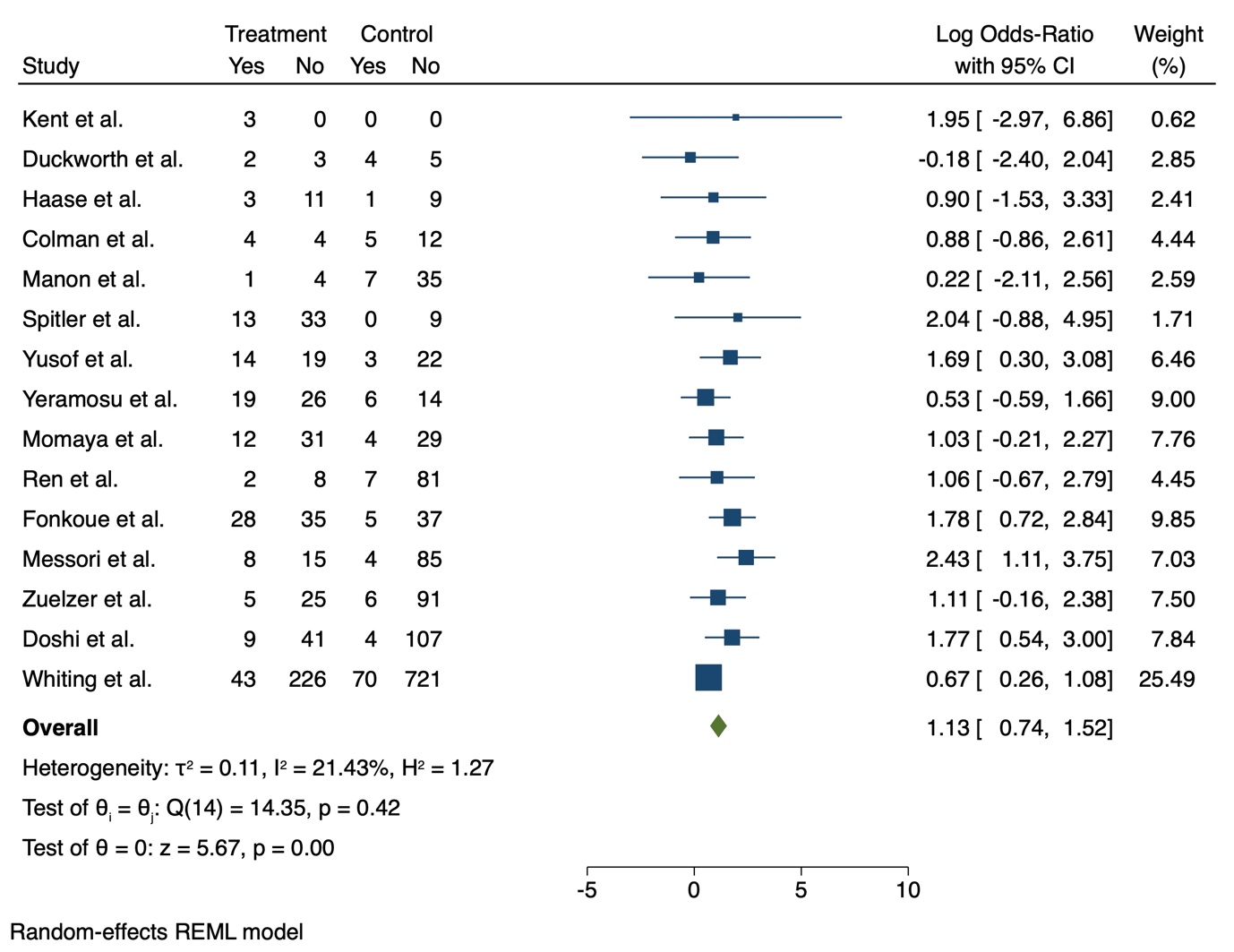


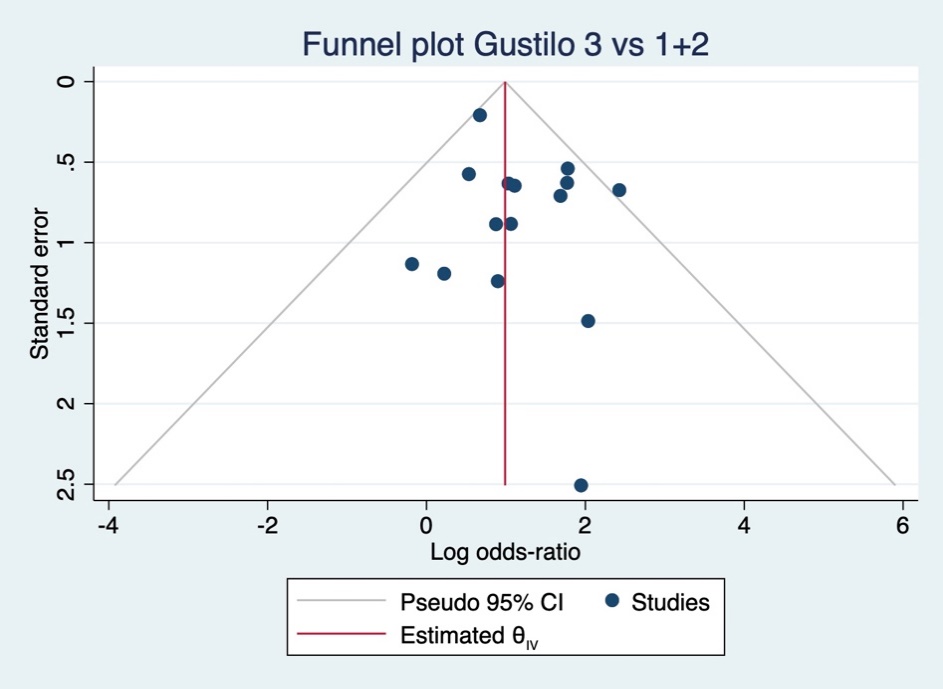


ASA


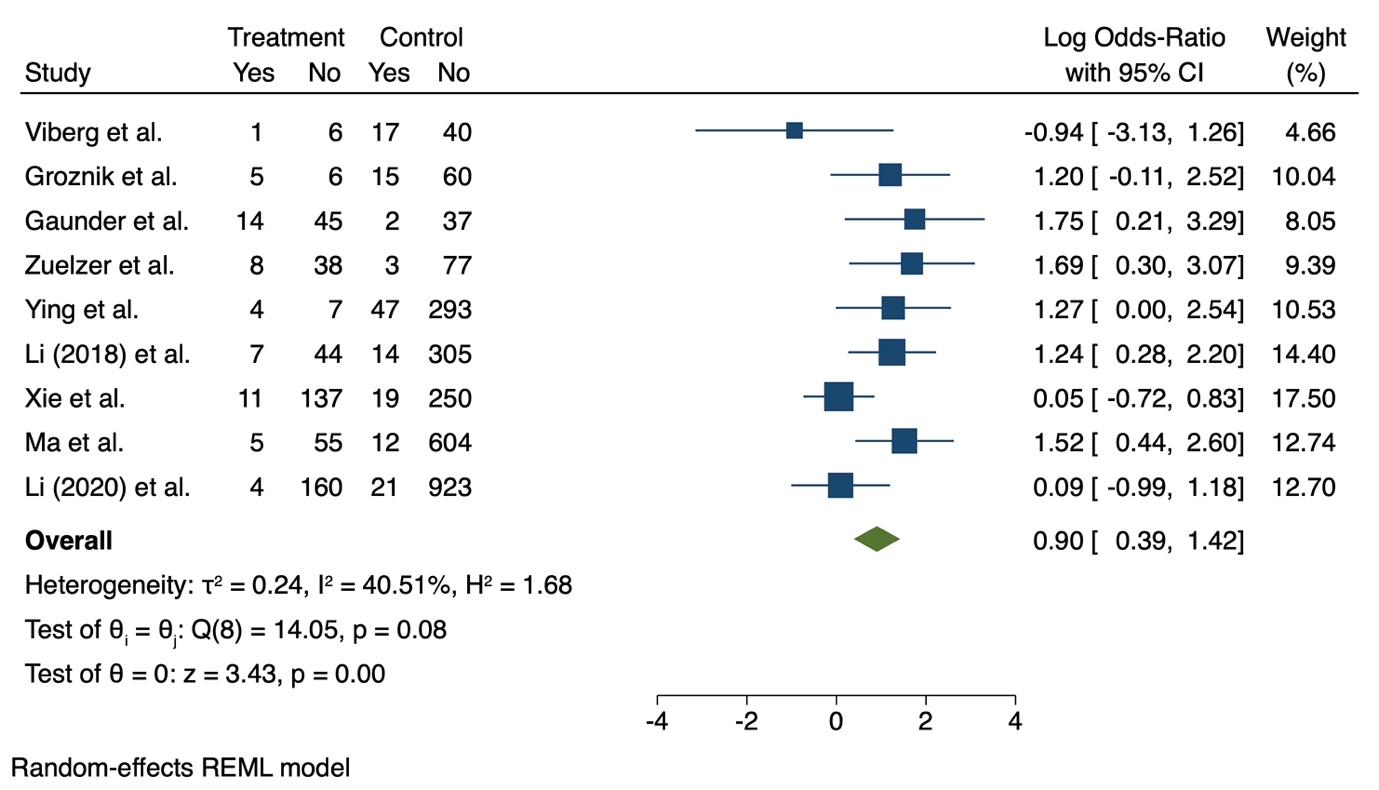


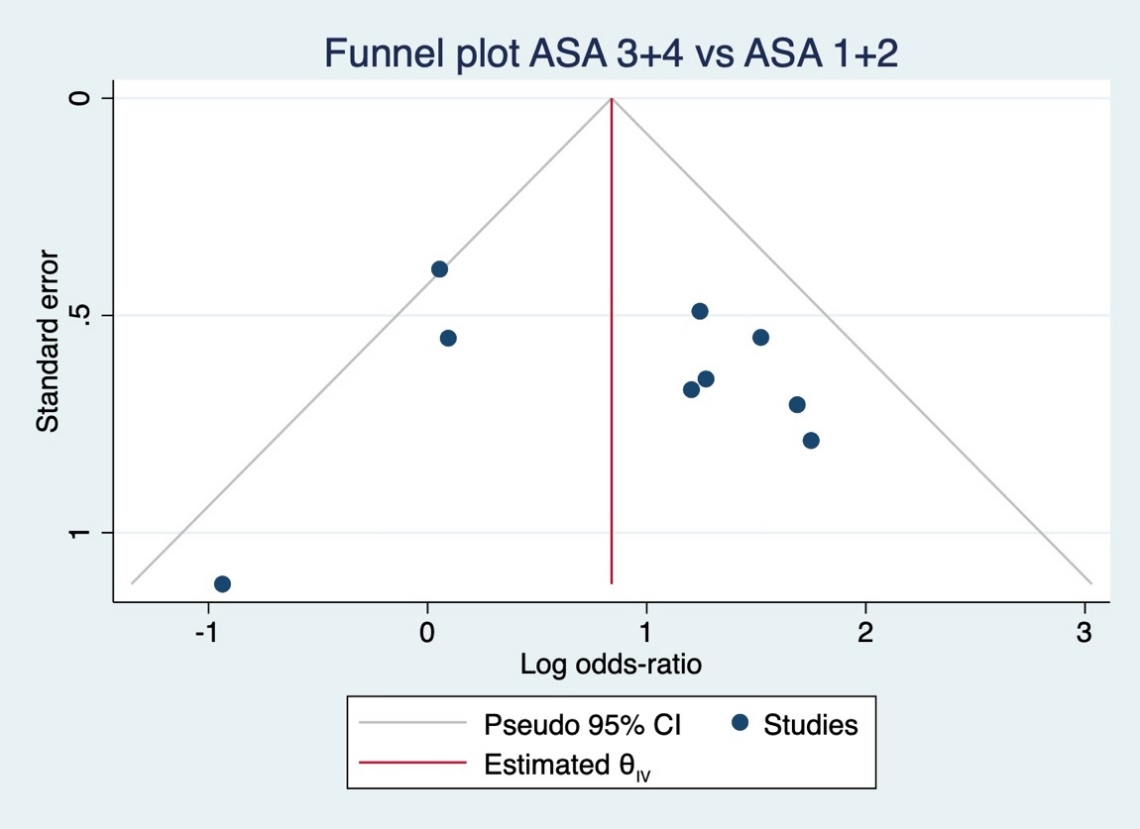


Compartment syndrom


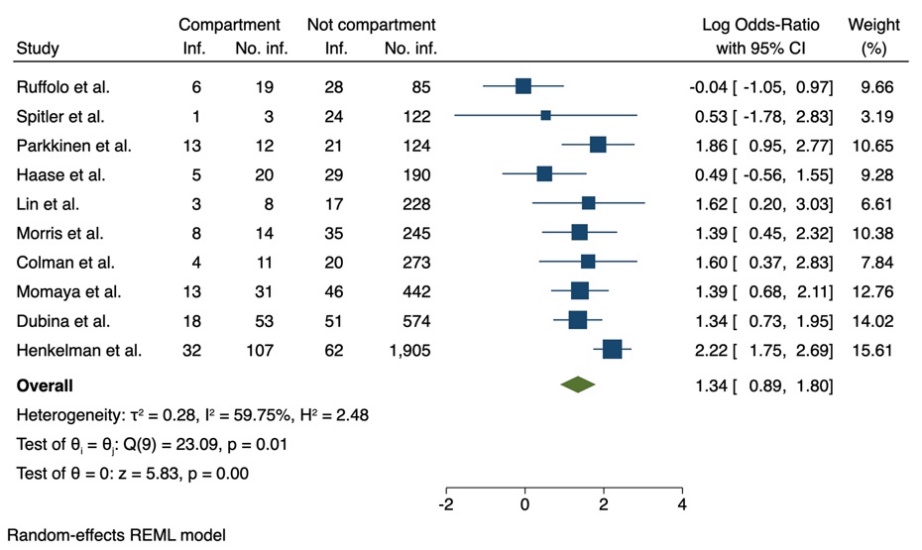


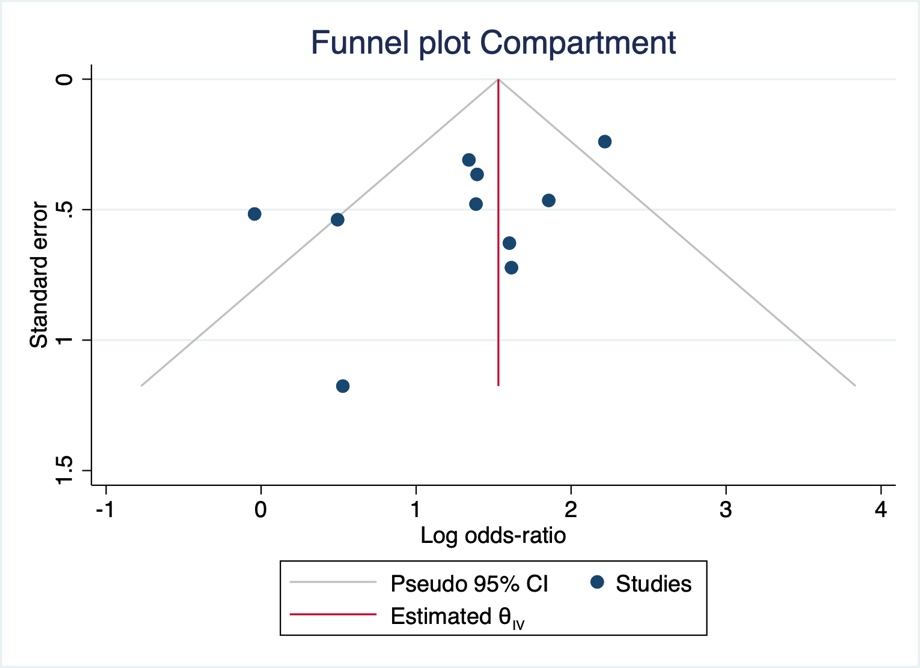


Polytrauma


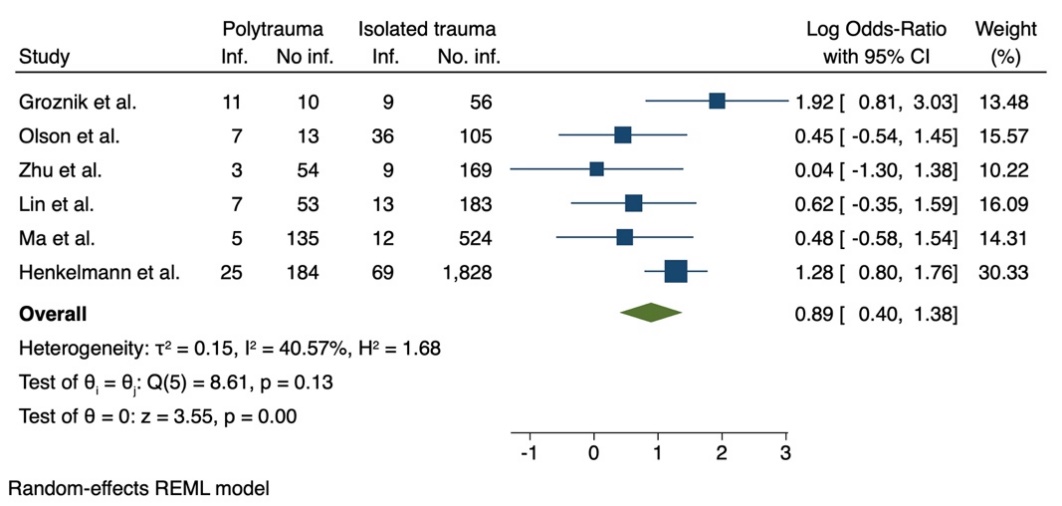


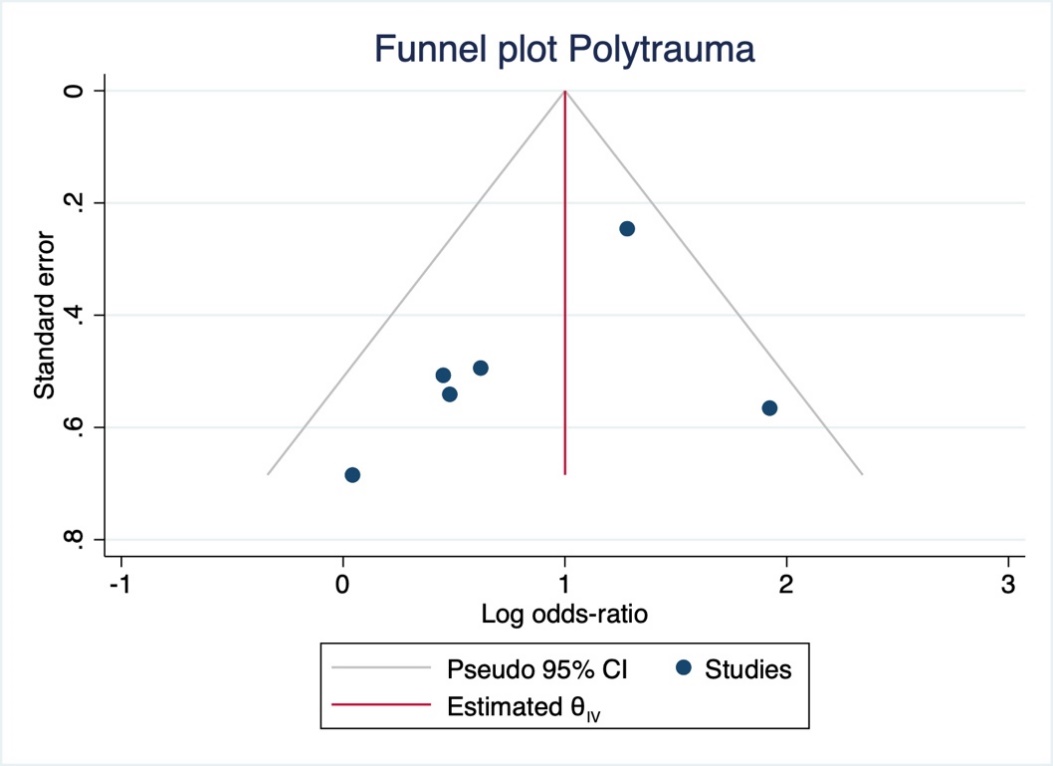


High Energy Trauma (H.E.T)


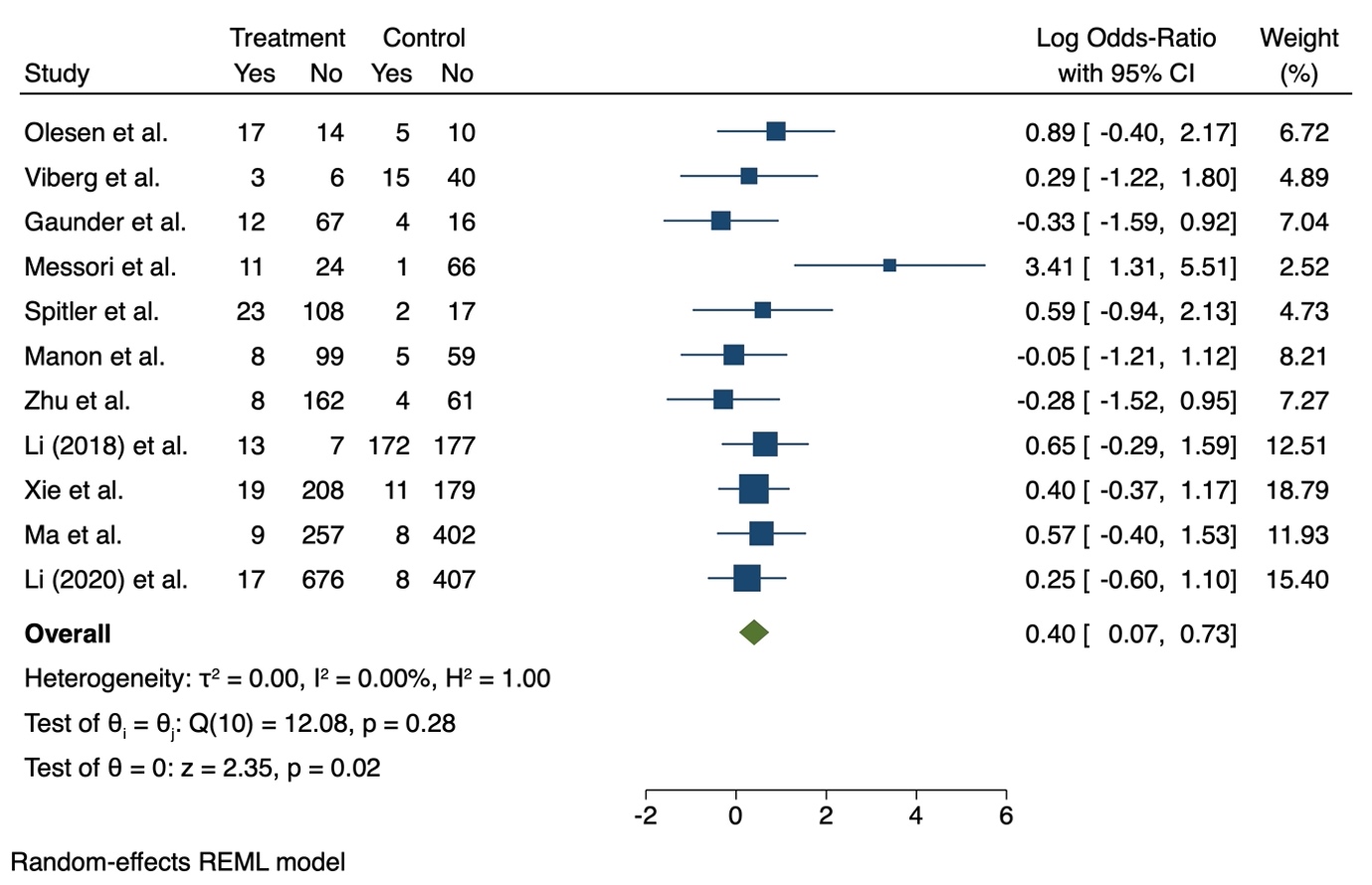


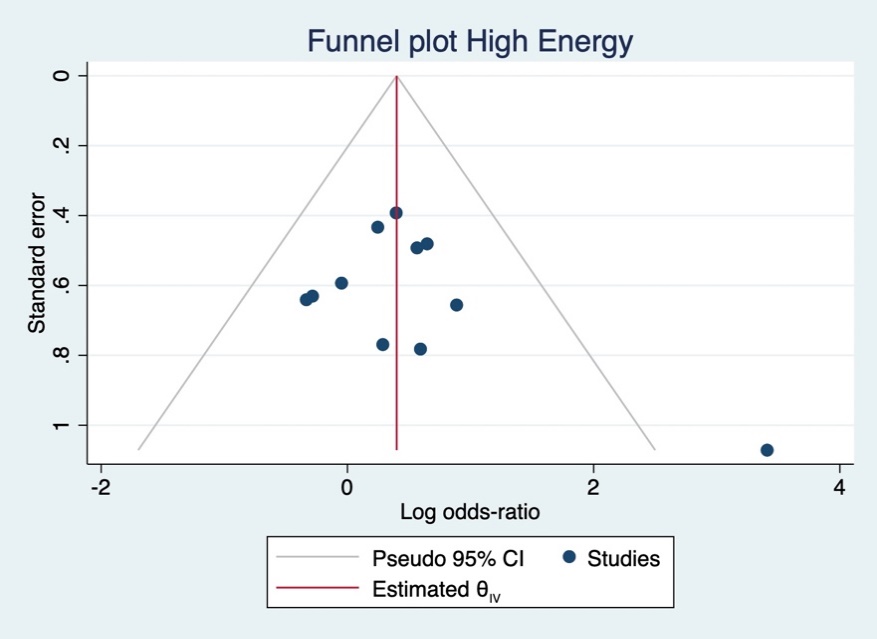


External fixation


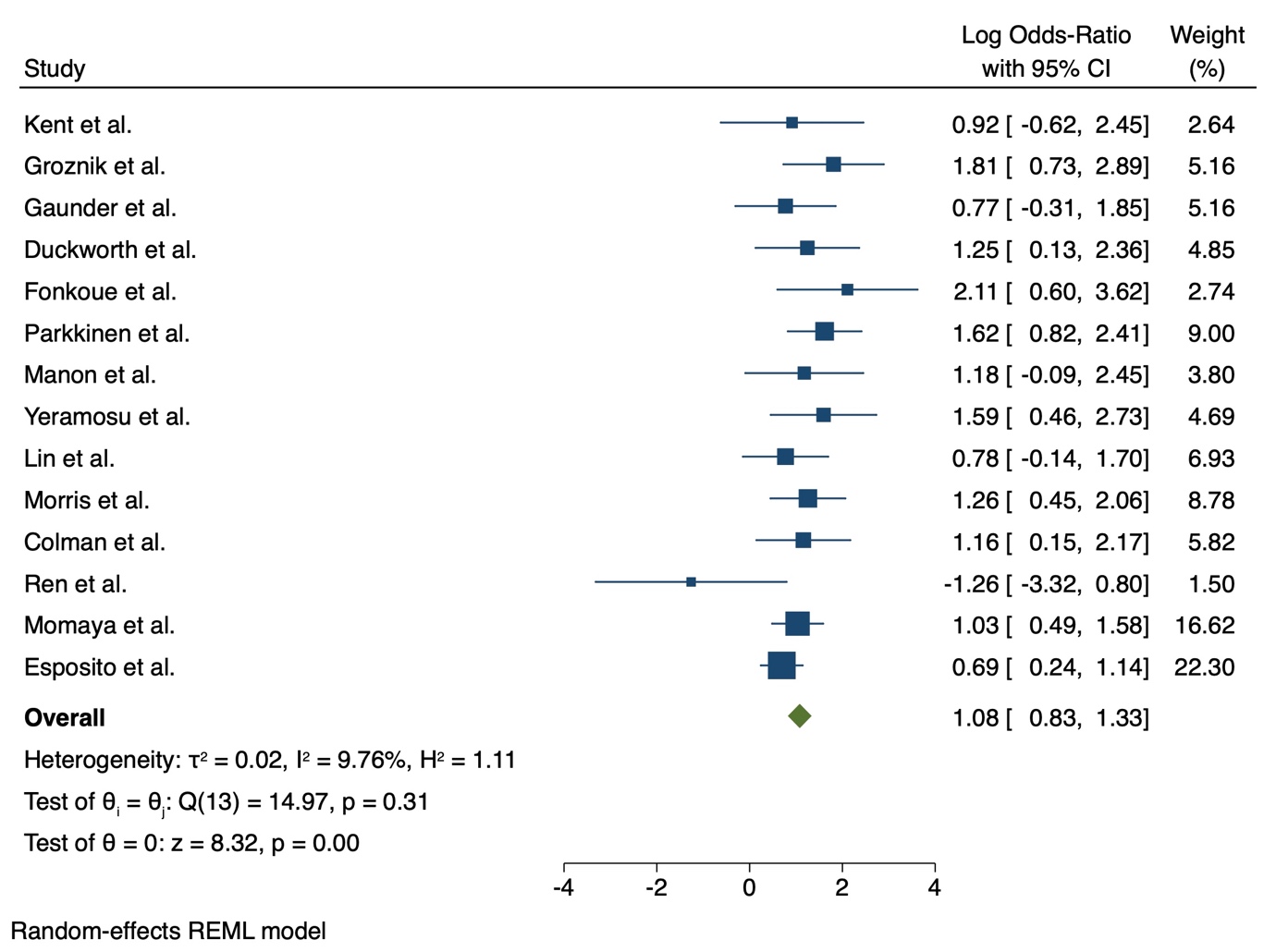


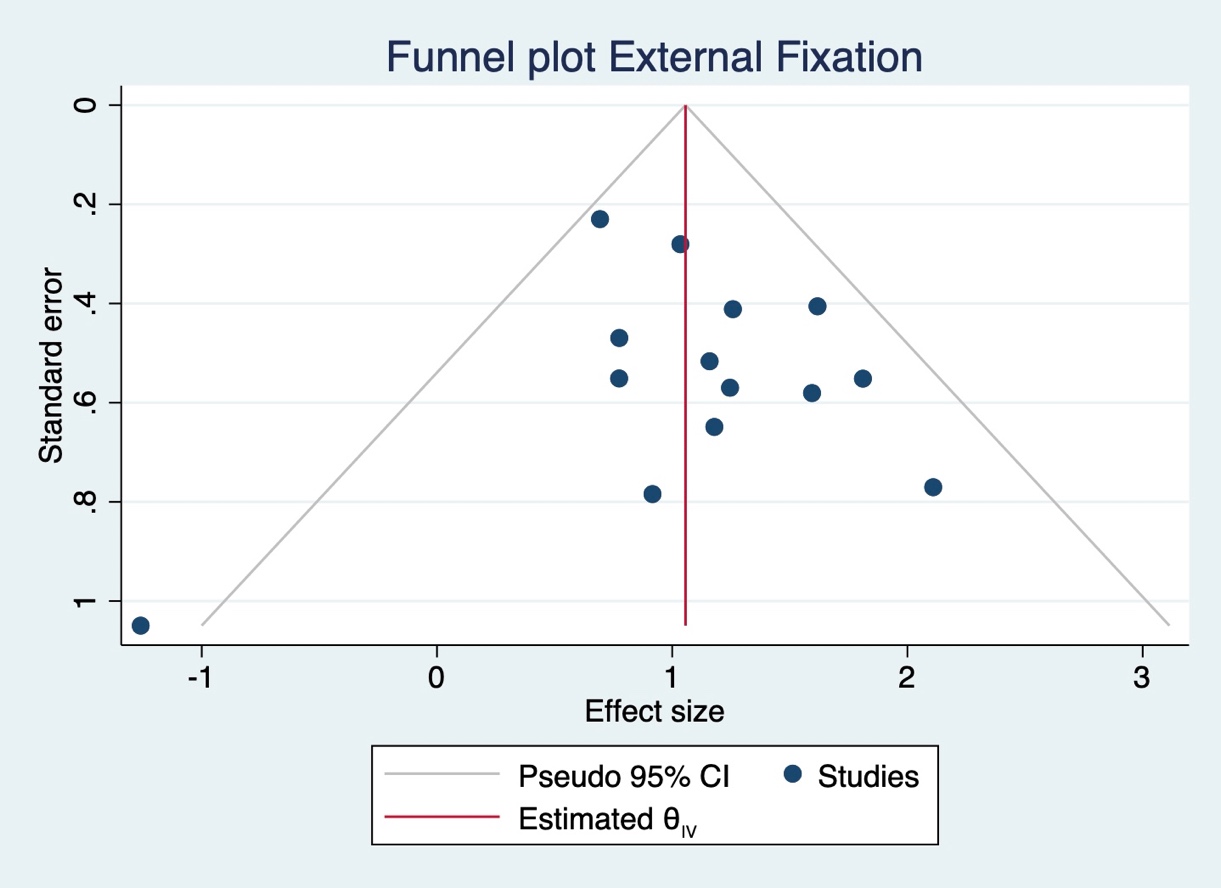


Dual incision approach


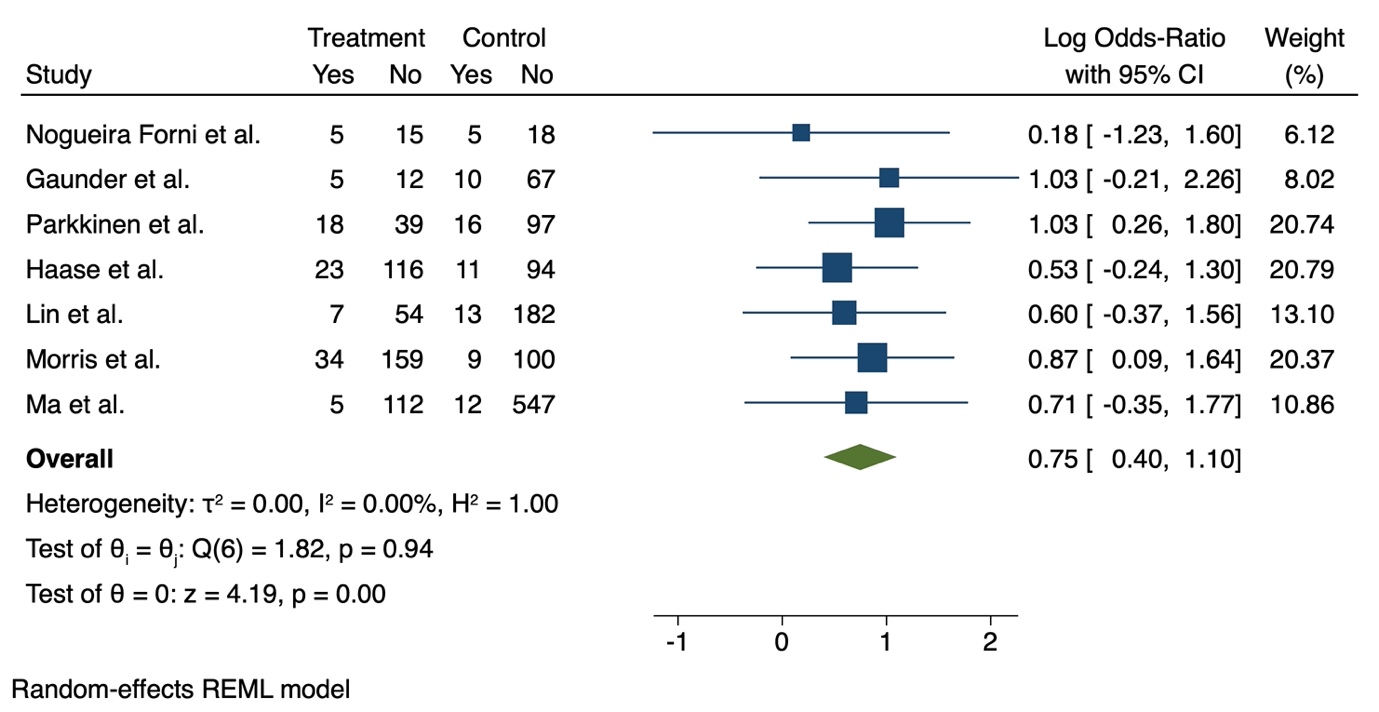


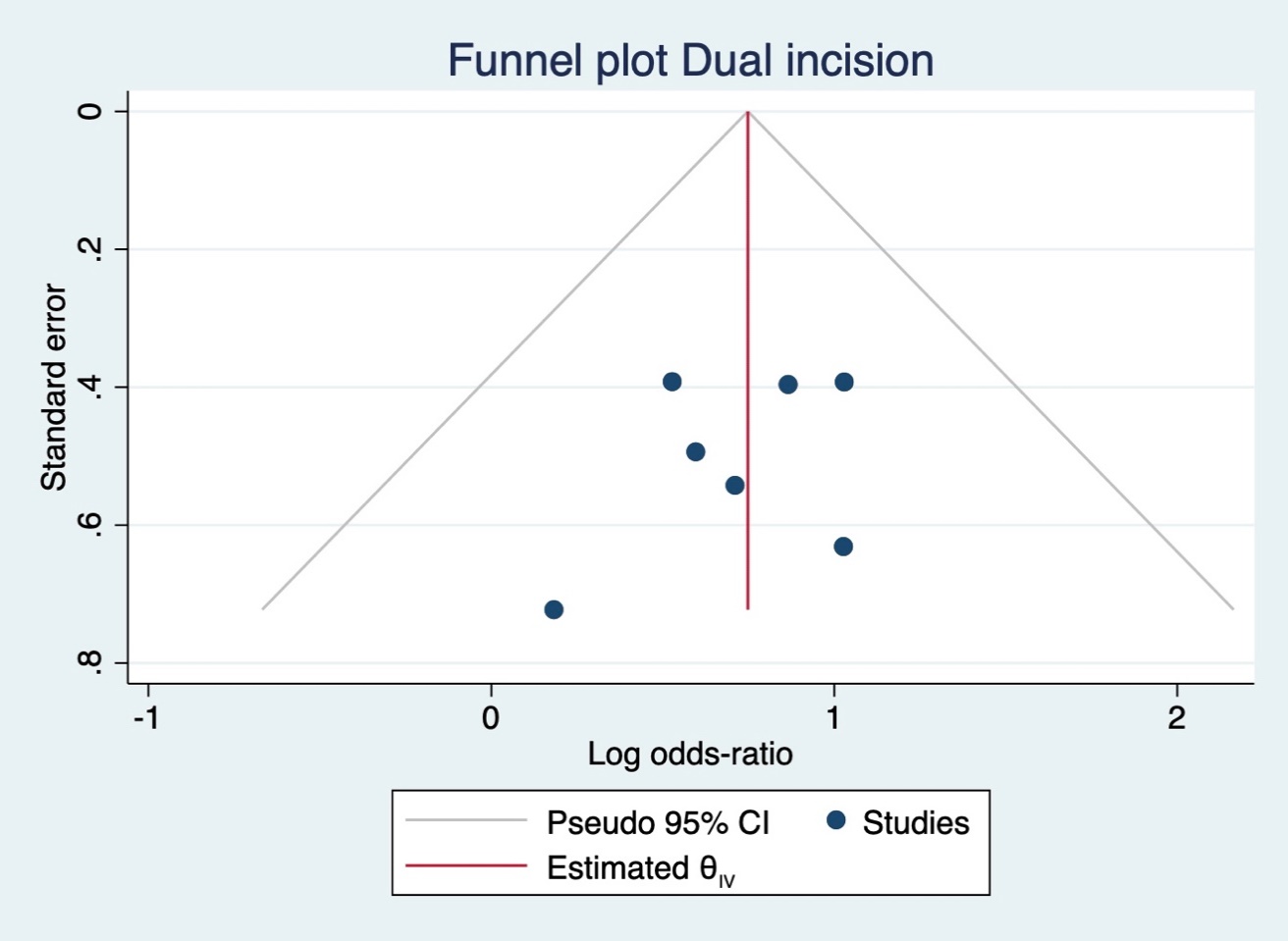


Time to surgery in days


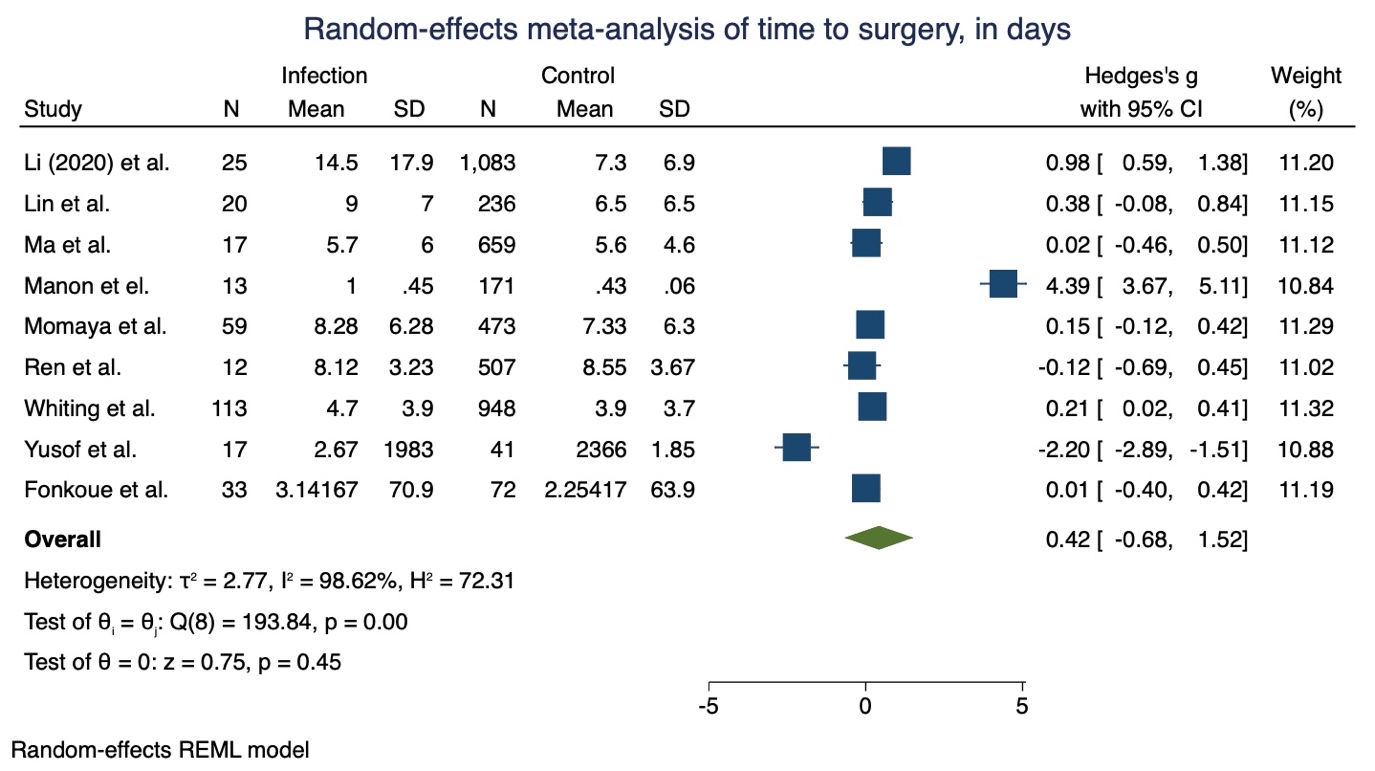


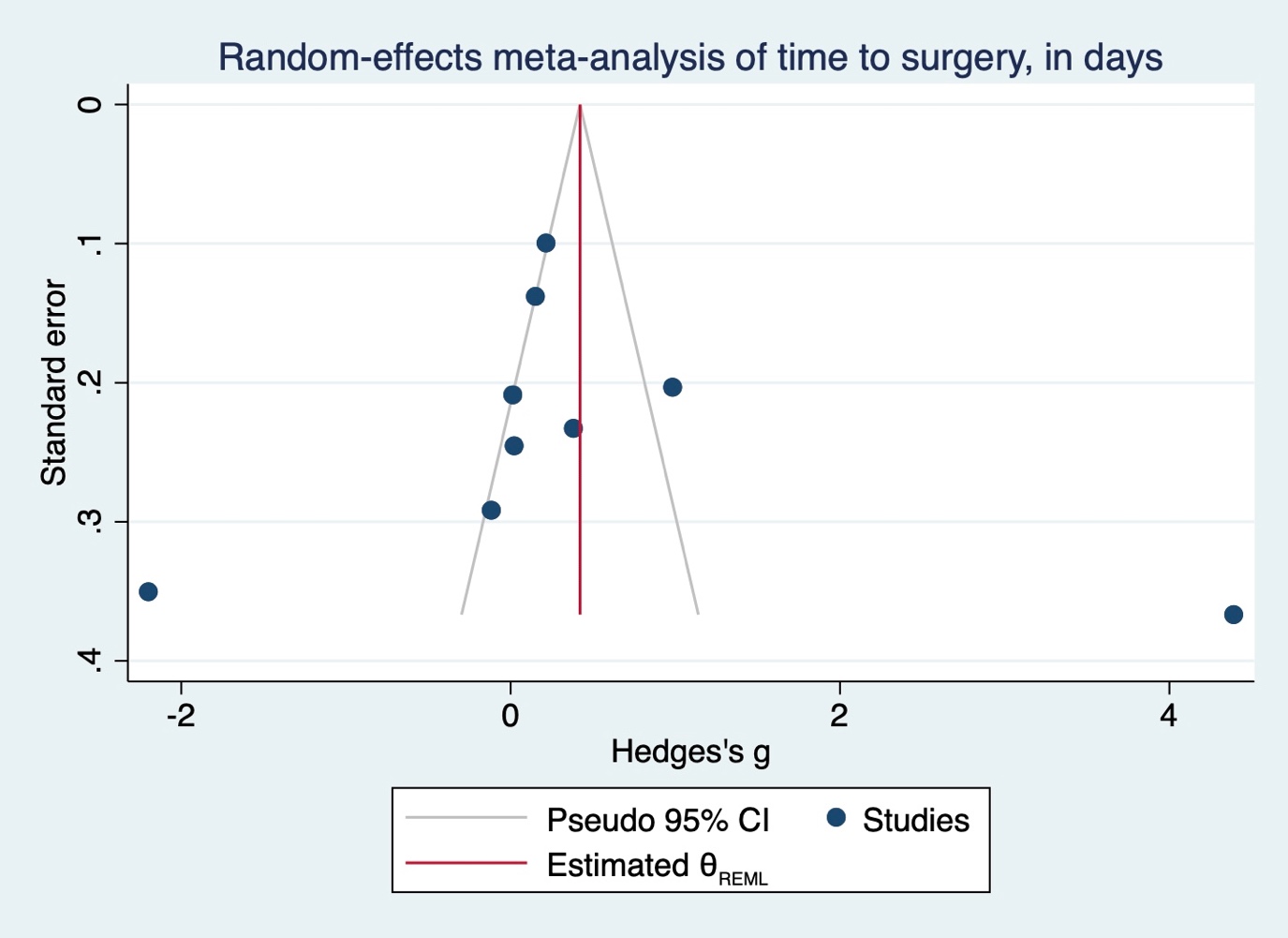


Mean operative time


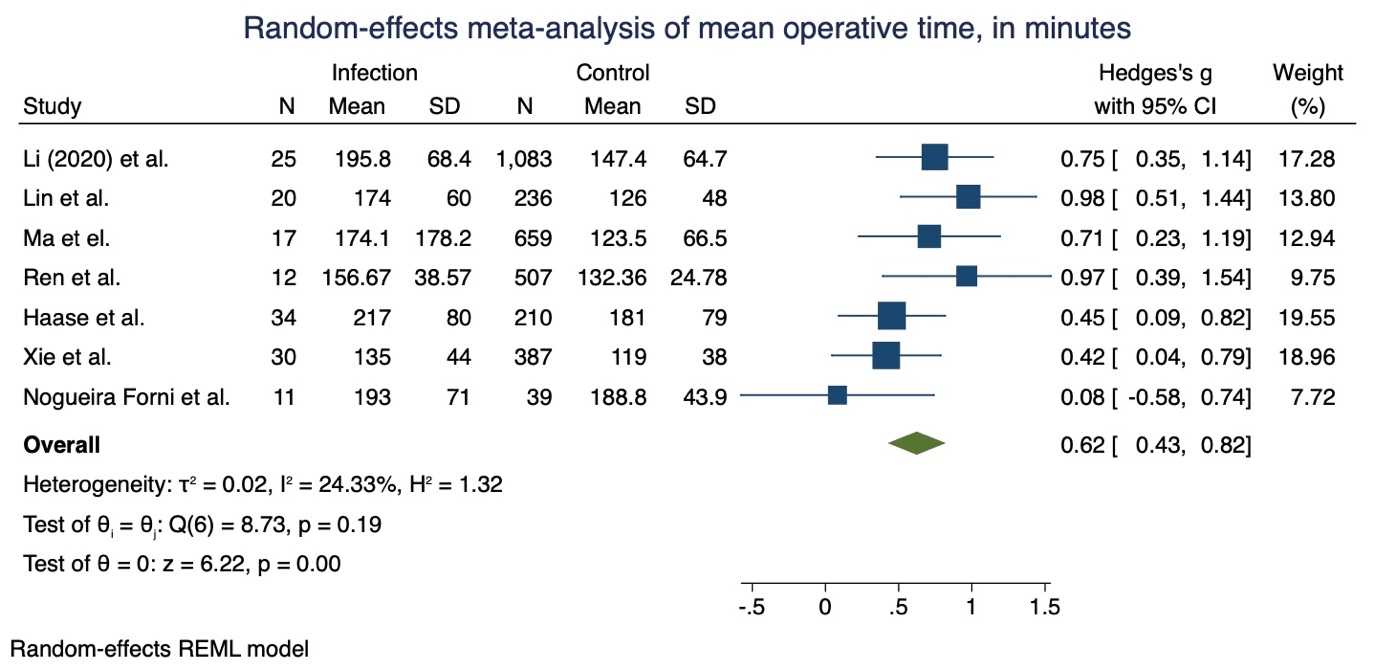


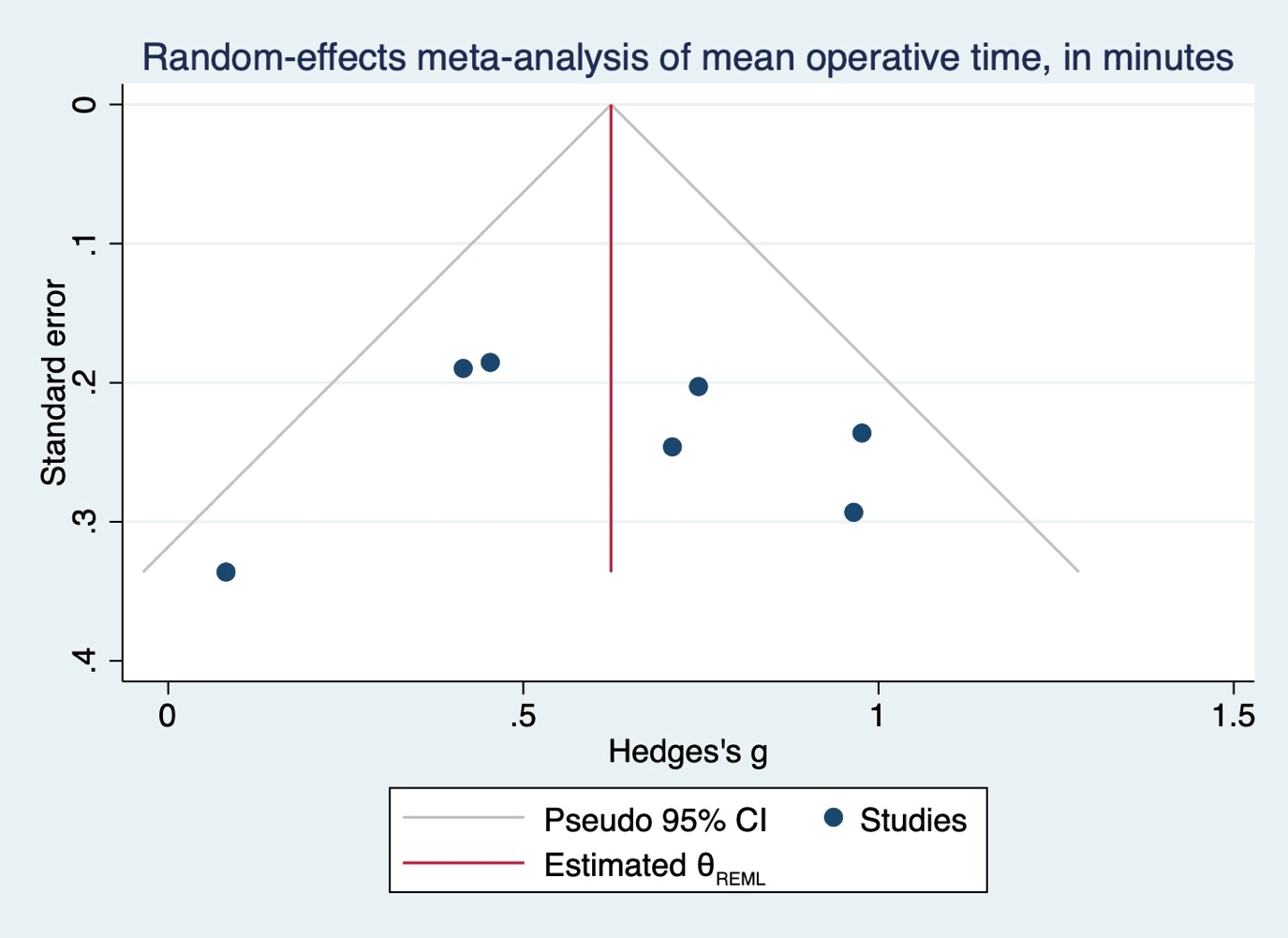


Blood transfusion


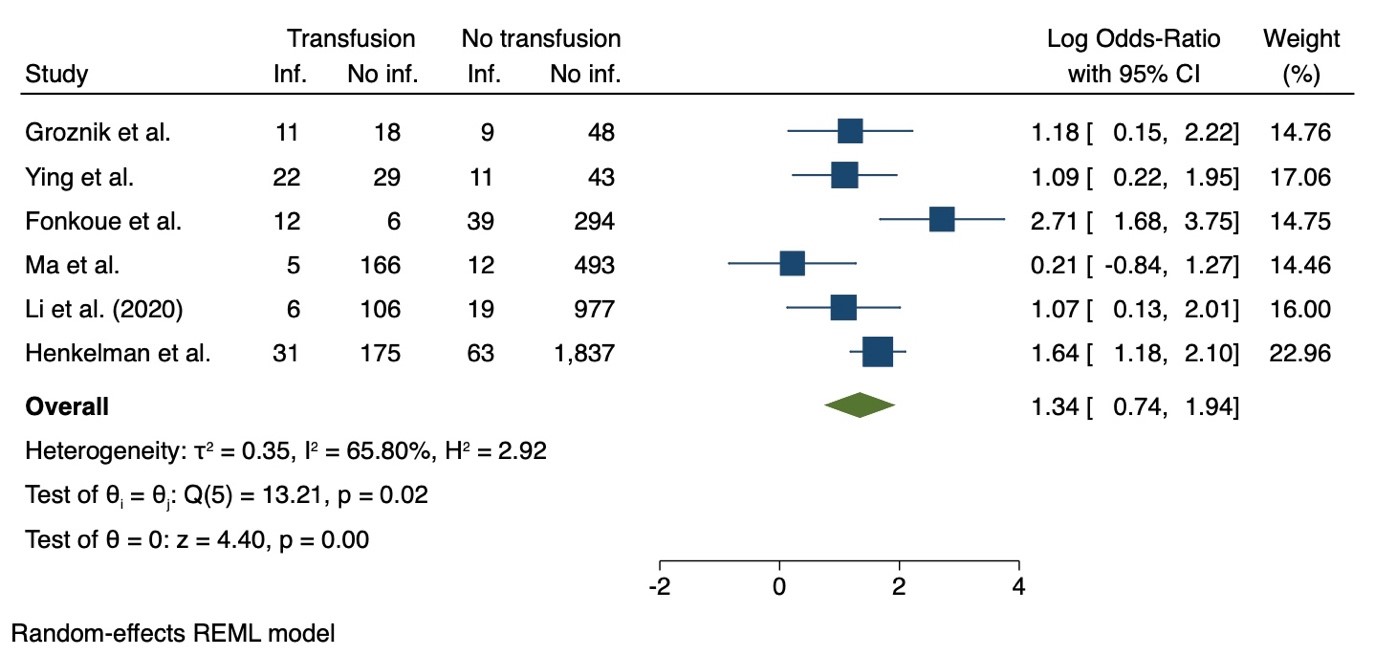


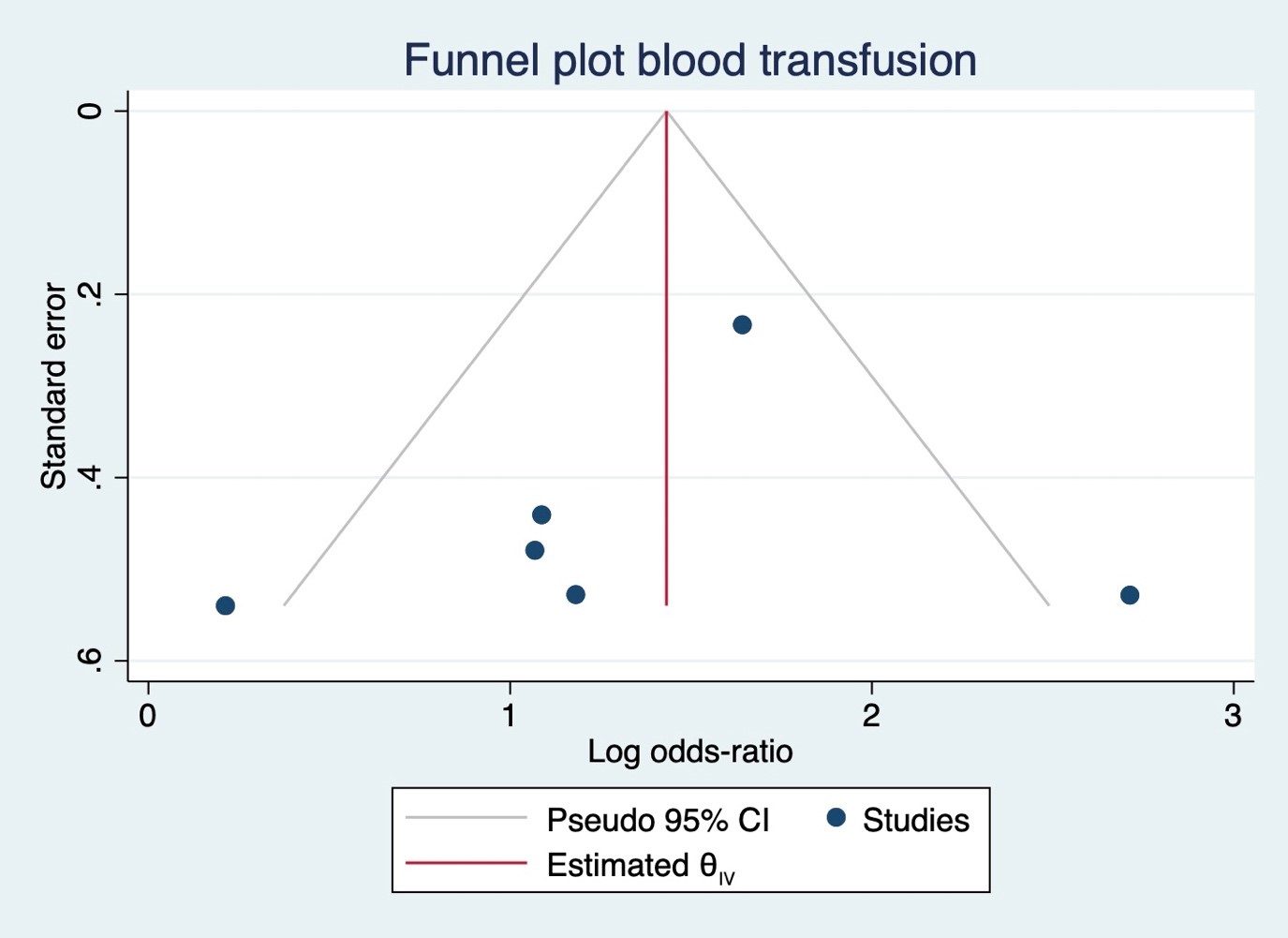

Supplement: Multimedia component 2 [file mmc2.docx]
