# Supplementary material for: Risk factors for tibial infections following osteosynthesis – a systematic review and meta-analysis
Source: J Clin Orthop Trauma. 2024 Feb 23;50:102376. doi: 10.1016/j.jcot.2024.102376 (PMC10909754; doi:10.1016/j.jcot.2024.102376)
Supplement: Multimedia component 3 [file mmc3.docx]

# 10. Appendix C

Fracture localization and risk factor distribution

| **Author** | **Male sex** | **Gustilo** | **Smoking** | **Diabetes** | **Open fracture** | **ASA classification** | **Polytrauma** | **Time to surgery** | **External fixation** | **Compartment** | **Hyper-tension** | **Dual incision approach** | **High energy trauma** | **Duration of surgery** |
| --- | --- | --- | --- | --- | --- | --- | --- | --- | --- | --- | --- | --- | --- | --- |
| **Fracture localization - Unspecified tibia** | | | | | | | | | | | | | | |
| Doshi et al. |  | 1 |  |  | 1 |  |  |  |  |  |  |  |  |  |
| Fonkoue et al. | 1 |  | 1 | 1 | 1 |  |  | 1 | 1 |  |  |  |  |  |
| Groznik et al. | 1 |  |  |  | 1 | 1 | 1 |  | 1 |  |  |  |  |  |
| Jenny et el. |  | 1 |  |  | 1 |  |  |  |  |  |  |  |  |  |
| Olesen et al. |  |  | 1 |  |  |  |  |  |  |  |  |  | 1 |  |
| Ying et al. | 1 |  | 1 | 1 |  | 1 |  |  |  |  | 1 |  |  | 1 |
| Yusof et al. | 1 | 1 |  |  |  |  |  |  |  |  |  |  |  |  |
| Zuelzer et al. | 1 | 1 | 1 | 1 |  | 1 |  | 1 |  |  |  |  |  |  |
| **Total number** | 5 | 4 | 4 | 3 | 4 | 3 | 1 | 2 | 2 |  | 1 |  | 1 | 1 |
| **Significant number** | 0 | 3 | 0 | 1 | 3 | 0 | 1 | 0 | 2 |  | 0 |  | 0 | 1 |
| **Fracture localization – Plateau/proximal tibia** | | | | | | | | | | | | | | |
| Colman et al. | 1 | 1 | 1 | 1 | 1 |  |  | 1 | 1 | 1 |  | 1 |  | 1 |
| Dubina et al. | 1 |  |  |  |  |  |  |  |  | 1 |  |  |  |  |
| Forni et al. |  |  | 1 | 1 | 1 |  |  |  |  |  |  | 1 |  | 1 |
| Gaunder et al. |  |  | 1 | 1 | 1 | 1 |  |  | 1 |  |  | 1 | 1 |  |
| Haase et al. |  | 1 |  |  | 1 |  |  |  |  | 1 |  | 1 |  | 1 |
| Henkelmann et al. | 1 |  | 1 | 1 | 1 |  | 1 |  |  | 1 |  |  |  |  |
| Li et al. (2018) | 1 |  | 1 | 1 | 1 | 1 | 1 | 1 |  |  | 1 |  | 1 | 1 |
| Li et al. (2020) |  |  | 1 | 1 |  | 1 |  | 1 |  |  | 1 |  | 1 | 1 |
| Lin et al. | 1 |  | 1 | 1 | 1 |  | 1 | 1 | 1 | 1 |  | 1 |  | 1 |
| Ma et al. |  |  | 1 | 1 |  | 1 | 1 | 1 |  |  | 1 | 1 | 1 | 1 |
| Momaya et al. | 1 | 1 | 1 | 1 | 1 |  |  | 1 | 1 | 1 |  |  |  |  |
| Morris et al. | 1 |  | 1 | 1 | 1 |  |  |  | 1 | 1 |  | 1 |  |  |
| Parkkinen et al. |  |  | 1 |  | 1 | 1 |  | 1 | 1 | 1 |  | 1 |  | 1 |
| Ruffolo et al. |  |  | 1 | 1 | 1 |  |  |  |  | 1 |  |  |  |  |
| Zhu et al. | 1 |  | 1 | 1 | 1 |  | 1 | 1 |  |  | 1 |  | 1 | 1 |
| Kugelman et al. |  |  |  |  | 1 |  |  |  |  | 1 |  |  |  |  |
| **Total number** | 8 | 3 | 13 | 12 | 13 | 5 | 5 | 8 | 6 | 10 | 4 | 8 | 5 | 9 |
| **Significant number** | 3 | 2 | 6 | 1 | 11 | 3 | 1 | 2 | 4 | 8 | 0 | 3 | 0 | 7 |
| **Fracture localization - Shaft** | | | | | | | | | | | | | | |
| Burrus et al. |  |  |  |  |  |  |  |  |  |  |  |  |  |  |
| Manon et al. | 1 | 1 | 1 | 1 | 1 |  |  | 1 | 1 |  |  |  | 1 |  |
| Whiting et al. | 1 | 1 |  |  |  |  |  | 1 |  |  |  |  |  |  |
| Metsemaker et al. |  | 1 | 1 | 1 | 1 | 1 | 1 |  | 1 |  |  |  |  |  |
| **Total number** | 2 | 3 | 2 | 2 | 2 | 1 | 1 | 2 | 2 |  |  |  | 1 |  |
| **Significant number** | 0 | 1 | 0 | 0 | 1 | 0 | 0 | 2 | 1 |  |  |  | 0 |  |
| **Fracture localization – Pilon/distal tibia** | | | | | | | | | | | | | | |
| Ashworth et al. | 1 |  | 1 | 1 | 1 |  |  | 1 |  |  |  |  |  |  |
| Duckworth et al. |  | 1 |  |  | 1 |  |  |  | 1 |  |  |  |  |  |
| Esposito et al. | 1 |  | 1 | 1 | 1 |  |  |  | 1 |  |  | 1 |  |  |
| Kent et al. | 1 | 1 | 1 |  | 1 |  |  |  | 1 |  |  |  |  |  |
| Kline et al. |  |  |  | 1 |  |  |  |  |  |  |  |  |  |  |
| Messori et al. | 1 |  |  | 1 | 1 |  |  |  |  |  |  |  | 1 |  |
| Molina et al. | 1 |  | 1 | 1 | 1 |  |  |  |  |  | 1 |  |  |  |
| Oladeji et al. |  |  |  | 1 |  |  |  |  |  |  |  |  |  |  |
| Olson et al. | 1 | 1 | 1 | 1 |  |  | 1 |  |  |  |  |  |  |  |
| Ren et al. |  | 1 | 1 |  | 1 |  |  | 1 | 1 |  |  |  |  | 1 |
| Spitler et al. | 1 | 1 | 1 | 1 | 1 |  |  |  |  | 1 |  |  | 1 |  |
| Viberg et al. |  |  | 1 |  | 1 | 1 |  |  |  |  |  |  | 1 |  |
| Xie et al. | 1 |  | 1 | 1 |  | 1 |  |  |  |  | 1 |  | 1 | 1 |
| Yeramosu et al. | 1 | 1 | 1 | 1 | 1 |  |  | 1 | 1 |  |  |  |  |  |
| **Total number** | 9 | 6 | 10 | 9 | 9 | 2 | 1 | 3 | 5 | 1 | 2 | 1 | 4 | 1 |
| **Significant number** | 3 | 5 | 3 | 4 | 6 | 0 | 0 | 0 | 3 | 0 | 1 | 0 | 0 | 1 |

Green color depicts significant results and yellow indicates that relevant statistics were not performed.
